# FUNDAMANT: an interventional 72-week phase 1 follow-up study of AADvac1, an active immunotherapy against tau protein pathology in Alzheimer’s disease

**DOI:** 10.1186/s13195-018-0436-1

**Published:** 2018-10-24

**Authors:** Petr Novak, Reinhold Schmidt, Eva Kontsekova, Branislav Kovacech, Tomas Smolek, Stanislav Katina, Lubica Fialova, Michal Prcina, Vojtech Parrak, Peter Dal-Bianco, Martin Brunner, Wolfgang Staffen, Michael Rainer, Matej Ondrus, Stefan Ropele, Miroslav Smisek, Roman Sivak, Norbert Zilka, Bengt Winblad, Michal Novak

**Affiliations:** 1 Axon Neuroscience CRM Services SE, Dvorakovo nabrezie 11, 811 02 Bratislava, Slovakia; 2 Clinical Division of Neurogeriatrics and Division of General Neurology, Department of Neurology, Medical University Graz, Auenbruggerplatz 2, 8036 Graz, Austria; 3 Axon Neuroscience R&D Services SE, Dvorakovo nabrezie 10, 811 02 Bratislava, Slovakia; 4 University Clinic of Neurology, Medical University of Vienna, Währinger Gürtel 18-20, 1090 Vienna, Austria; 5 University Clinic of Clinical Pharmacology, Medical University of Vienna, Währinger Gürtel 18-20, 1090 Vienna, Austria; 6 University Clinic of Neurology, Christian-Doppler-Clinic, Ignaz-Harrer-Straße 79, 5020 Salzburg, Austria; 7 Social and Medical Centre East, Danube Hospital, Karl Landsteiner Institute for Memory and Alzheimer Research, Langobardenstraße 122, 1220 Vienna, Austria; 8 Division of Neurogeriatrics, Department NVS Clinical Trial Unit, Karolinska Institute Alzheimer Disease Research Centre, Geriatric Clinic, Karolinska University Hospital, Hälsovägen 7, S-14157 Huddinge, Sweden; 9 Axon Neuroscience SE, 4, Arch. Makariou & Kalogreon, Nicolaides Sea View City, 5th floor, office 506, 6016 Larnaca, Cyprus

**Keywords:** Tau, Alzheimer’s disease, Vaccine, Immunotherapy, Active immunotherapy, Clinical trial, Neurofibrillary pathology

## Abstract

**Background:**

Neurofibrillary pathology composed of tau protein is closely correlated with severity and phenotype of cognitive impairment in patients with Alzheimer’s disease and non-Alzheimer’s tauopathies. Targeting pathological tau proteins via immunotherapy is a promising strategy for disease-modifying treatment of Alzheimer’s disease. Previously, we reported a 24-week phase 1 trial on the active vaccine AADvac1 against pathological tau protein; here, we present the results of a further 72 weeks of follow-up on those patients.

**Methods:**

We did a phase 1, 72-week, open-label study of AADvac1 in patients with mild to moderate Alzheimer’s disease who had completed the preceding phase 1 study. Patients who were previously treated with six doses of AADvac1 at monthly intervals received two booster doses at 24-week intervals. Patients who were previously treated with only three doses received another three doses at monthly intervals, and subsequently two boosters at 24-week intervals. The primary objective was the assessment of long-term safety of AADvac1 treatment. Secondary objectives included assessment of antibody titres, antibody isotype profile, capacity of the antibodies to bind to AD tau and AADvac1, development of titres of AADvac1-induced antibodies over time, and effect of booster doses; cognitive assessment via 11-item Alzheimer’s Disease Assessment Scale cognitive assessment (ADAS-Cog), Category Fluency Test and Controlled Oral Word Association Test; assessment of brain atrophy via magnetic resonance imaging (MRI) volumetry; and assessment of lymphocyte populations via flow cytometry.

**Results:**

The study was conducted between 18 March 2014 and 10 August 2016. Twenty-six patients who completed the previous study were enrolled. Five patients withdrew because of adverse events. One patient was withdrawn owing to noncompliance. The most common adverse events were injection site reactions (reported in 13 [50%] of vaccinated patients). No cases of meningoencephalitis or vasogenic oedema were observed. New micro-haemorrhages were observed only in one ApoE4 homozygote. All responders retained an immunoglobulin G (IgG) antibody response against the tau peptide component of AADvac1 over 6 months without administration, with titres regressing to a median 15.8% of titres attained after the initial six-dose vaccination regimen. Booster doses restored previous IgG levels. Hippocampal atrophy rate was lower in patients with high IgG levels; a similar relationship was observed in cognitive assessment.

**Conclusions:**

AADvac1 displayed a benign safety profile. The evolution of IgG titres over vaccination-free periods warrants a more frequent booster dose regimen. The tendency towards slower atrophy in MRI evaluation and less of a decline in cognitive assessment in patients with high titres is encouraging. Further trials are required to expand the safety database and to establish proof of clinical efficacy of AADvac1.

**Trial registration:**

The studies are registered with the EU Clinical Trials Register and ClinicalTrials.gov: the preceding first-in-human study under EudraCT 2012-003916-29 and NCT01850238 (registered on 9 May 2013) and the follow-up study under EudraCT 2013-004499-36 and NCT02031198 (registered 9 Jan 2014), respectively.

**Electronic supplementary material:**

The online version of this article (10.1186/s13195-018-0436-1) contains supplementary material, which is available to authorized users.

## Background

Alzheimer’s disease (AD) is a primary progressive neurodegenerative disorder with an insidious course that is initially clinically silent, then manifests as subtle cognitive impairments, and finally blossoms into full-fledged dementia that deprives the patient of memory, orientation, judgement and language, leaving them wholly dependent on care. The clinically silent phase can take decades, whereas the symptomatic phase runs its course over 5–10 years [1, 2].

Being an age-associated disorder, AD is becoming more common as medical advances across disciplines extend the human life-span [1]. Currently approved medications are only able to compensate for neurotransmitter loss and do not halt the underlying disease process [3]. Besides the ultimately lethal nature of AD, its protracted disease course and care dependence of patients place an immense strain on caregivers and healthcare systems [4]; for all of the above reasons, the development of disease-modifying AD therapies is of utmost importance.

AD is a multifactorial disorder, and the field of disease-modifying therapies is thus necessarily diverse [5]. Based on the two dominant brain protein pathologies [6]—plaques and arterial deposits consisting of amyloid-β, and neurofibrillary pathology composed of protein tau—two leading hypotheses emerged. The amyloid cascade hypothesis is supported by the fact that mutations in the amyloid precursor protein and the presenilins involved in its processing can cause AD in an autosomal dominant fashion [7]; the tau hypothesis draws its support from the observation that brain atrophy and cognitive and functional impairment in AD are directly tied to the amount and localisation of tau pathology [2, 6, 8]. Furthermore, neurofibrillary pathology alone is fully capable of causing a range of neurodegenerative disorders fittingly termed *tauopathies* [9, 10]. In health, tau protein is intimately associated with microtubule dynamics, neuronal plasticity and axonal transport [11]. In disease, through loss of function and toxic gain of function, pathological tau protein leads to synaptic damage, reduced neuronal plasticity, microtubule destabilisation and neuronal death [12].

The development of disease-modifying therapies against AD pathologies has taken both the small-molecule and immunotherapy routes [13]. Active immunotherapies possess a range of highly attractive attributes. The most obvious is the potential to be used preventively if shown to be efficacious at halting or slowing down the progression of dementia, an approach which is not as feasible with monoclonal antibodies (mAbs) [14]. Vaccines in general are one of the most cost-effective yet impactful medical interventions; treatment costs between active immunisation and mAbs differ by orders of magnitude. Additionally, by using the patient’s immune system to produce antibodies, active immunisation avoids challenges such as anti-drug antibodies that plague humanised mAbs [15]. Beside their benefits, active immunotherapies also face unique challenges, as evidenced by the autoimmune meningoencephalitis caused by the anti-amyloid vaccine AN1792 [16]. The initial reaction was the development of a range of mAbs [3], but second-generation active vaccines such as AADvac1 [17] or CAD106 [18] showed that targeting of pathological proteins in AD can be performed safely, without evoking a self-directed T-cell response. Despite intense efforts, efficacy has not yet been proven for any disease-modifying therapy for AD.

We have previously reported creation of AADvac1, an active vaccine that would elicit an immune response against an epitope that is a common, functionally important denominator of tau pathology [19]. Both active (AADvac1) and passive (DC8E8) immunotherapy resulted in the improvement of neurobehavioral impairment of transgenic animals, as well as reduction in neurofibrillary pathology and sarkosyl-insoluble tau protein in their brains [19, 20]. AADvac1 was investigated in a first-in-human study in patients with mild to moderate AD dementia, with encouraging results in both safety and immunogenicity [17]. With AD being a chronic disorder, it is expected that potential therapies will likewise have to be applied over extended periods of time, and their effects (if they are applied in the symptomatic phase of AD) manifesting as change in the course of disease-driven decline. Therefore, we have conducted a follow-up study for the phase 1 trial of AADvac1, primarily to study long-term safety and development of the immune response after the initial vaccination regimen. Together, the data for the two studies cover 96 weeks of treatment.

### Research in context

Despite numerous setbacks, compounds based on the amyloid hypothesis (whether immunotherapies or small molecules) continue to be investigated vigorously, with focus shifting towards evaluating their efficacy in early and preclinical stages of AD (e.g., A4: Anti-Amyloid Treatment in Asymptomatic Alzheimer’s Disease [21] or the API: Alzheimer’s Prevention Initiative [22]).

Several other anti-tau immunotherapeutics are being tested in clinical trials. Three mAbs—C2N 8E12 (C2N; AbbVie, North Chicago, IL, USA) [23], RO 7105705 (AC Immune, Lausanne, Switzerland; Genentech, South San Francisco, CA, USA; Hoffmann-La Roche, Basel, Switzerland) and BMS-986168 (Biogen, Cambridge, MA, USA) [24]—recognise various epitopes on the N-terminus of tau protein. They target mostly extracellular N-terminal fragments and/or all six tau isoforms. The active liposome-based vaccine ACI-35 (AC Immune; Janssen, Beerse, Belgium) [25] is being tested in a phase 1 clinical trial. The vaccine generates antibodies recognising tau protein phosphorylated at pS396/pS404. The discontinued RG7345 passive vaccine similarly aimed for the C-terminus, specifically the phospho-epitope pS422 [26].

In contrast to the aforementioned tau therapeutic strategies, AADvac1 induces generation of antibodies targeting a part of the tau molecule that is preserved in all aggregating truncated tau species: the microtubule-binding repeat domain (MTBR). Some newly introduced tau-targeted immunotherapies seem to follow this rationale (LY3303560, UCB1017) (*see also* www.alzforum.org). Little has been reported to date about other recently introduced compounds (JNJ-63733657, BIIB076) [27].

## Methods

### Study design

The present study (protocol AC-AD-002) was a 72-week, open-label, single-arm interventional follow-up trial for the 24-week first-in-human study of AADvac1 (protocol AxonCO18700) [17]. The study was conducted in four centres in Austria (University Hospital Graz; Medical University Wien; Social and Medical Centre East, Danube Hospital Wien; and Christian-Doppler Clinic Salzburg).

### Patients

The study enrolled only patients who had completed the preceding phase 1 study of AADvac1. Thus, the inclusion/exclusion criteria were minimal, requiring informed consent capability; the availability of a caregiver; and the absence of severe co-morbidities, immunosuppressive treatment, or contraindications for magnetic resonance imaging (MRI).

The preceding study enrolled patients aged 50–85 years with a diagnosis of Alzheimer’s dementia based on the National Institute of Neurological and Communicative Disorders and Stroke-Alzheimer’s Disease and Related Disorders Association criteria, a screening MRI study compatible with the diagnosis of AD, and a Mini Mental State Examination (MMSE) score of 15–26 (see Table 1 for demographic characteristics of the study population). *See* Additional file 1 for verbatim wording of the inclusion criteria of this study and the preceding first-in-human trial.

**Table 1:** Demographic characteristics

AC-AD-002 demography	*n* = 26
Age (years), mean (SD)	67.1 (8.1)
Education level (years), mean (SD)	12.0 (3.7)
Sex	
Male	16 (62%)
Female	10 (38%)
Ethnic origin	
Caucasian	26 (100%)
MMSE score (before treatment start), mean (SD)	20.7 (4.1)
Modified Hachinski score (before treatment start), mean (SD)	1.0 (0.9)
GDS (before treatment start), mean (SD)	1.3 (1.3)
ApoE status	
Carrier	16 (62%)
Non-carrier	10 (38%)
Smoking habit	
Smoker	1 (3.8%)
Non-smoker	25 (96.2%)
Medication status	
Receiving standard AD medication	24 (92.3%)
Not receiving standard AD medication	2 (7.7%)

*Abbreviations: AD* Alzheimer’s disease, *GDS* Geriatric Depression Scale, *MMSE* Mini Mental State Examination

Data are mean (SD) or number (%). Values labelled as “before treatment start” were recorded at the start of the preceding study and not re-recorded at this study’s screening

### Investigational medicinal product

Each dose of AADvac1 consisted of 40 μg of Axon Peptide 108 (N-terminally cysteinylated tau 294–305/4R, amino acid sequence CKDNIKHVPGGGS) coupled to keyhole limpet haemocyanin (KLH) via a maleimide linker, with aluminium hydroxide adjuvant (containing 0.5 mg Al^3+^) in a phosphate buffer volume of 0.3 ml. The design of the vaccine has been described in detail previously [19].

The treatment regimen of the preceding study consisted of six doses of AADvac1 in 4-week intervals (three doses in the double-blind phase and three doses in the open-label phase). Patients randomised to placebo have instead received three doses of placebo in the double-blind phase, followed by three doses of AADvac1 in the open-label phase [17].

Patients assigned to AADvac1 in the double-blind phase of the preceding study have received two booster doses in 24-week intervals in this trial. The first booster dose was optional, to be administered if the titres of antibodies against Axon Peptide 108 declined below 75% of titres achieved following the initial six-dose vaccination regimen. The second booster dose was obligatory.

Patients assigned to placebo in the double-blind phase of the preceding study have first received three doses of AADvac1 in 4-week intervals (“catch-up visits”) to bring their vaccination regimen in line with patients who had received AADvac1 in the double-blind phase, and subsequently entered the above-described booster regimen.

Thus, in the two studies combined, both groups have received an identical AADvac1 treatment regimen: six doses administered in 4-week intervals, followed by two boosters in 24-week intervals. Both groups have a total of 96 weeks of on-treatment data and are pooled for analyses that measure on-treatment change. *See* Additional file 1 for an illustration.

### Safety evaluation

Reports of adverse events were obtained by non-directive questioning from patients and their caregivers, as well as by reviewing the patient diary. A structured neurological and physical examination was done by the investigators at 12-week intervals. Vital signs were assessed at every visit and 1 h after AADvac1 administration. Standard laboratory panels (biochemistry, coagulation, haematology, and inflammation markers) and dipstick urinalysis were assessed at 12-week intervals; a laboratory urinalysis was done if indicated by pathological dipstick results. Investigators reviewed laboratory results and reported clinically significant abnormalities as adverse events. A standard electrocardiogram was done every 24 weeks. Safety MRI was done every 24 weeks, and each scan was assessed in parallel by the investigator and the sponsor’s radiologist. Accruing safety data were reviewed every 6 months by an independent Data and Safety Monitoring Board (DSMB).

### Immunogenicity

An indirect enzyme-linked immunosorbent assay (ELISA) was used to measure the titres of vaccine-induced antibodies. Axon Peptide 108, tau151–391/4R, and KLH were used as solid phase, separately immobilised on microtitre plates (High Binding strip plates; Greiner Bio-One, Frickenhausen, Germany), and incubated overnight with serially diluted patient serum samples. After extensive washing, bound antibodies were detected by anti-human immunoglobulins conjugated to horseradish peroxidase (anti-human subclass-specific secondary antibodies for the detection of IgM and IgG, IgG subclasses IgG1, IgG2, IgG3 and IgG4, and anti-human IgA + IgG + IgM for anti-KLH antibodies; all provided by Pierce Biotechnology, Rockford, IL, USA). We measured the amounts of bound secondary antibodies through the activity of horseradish peroxidase with the ready-to-use chromogenic substrate TMB One (KEM-EN-TEC Diagnostic, Taastrup, Denmark) and the absorbance at 450 nm. The resulting signal was compared with that obtained for the patient’s serum collected at baseline.

We defined the titre of the antibodies in the serum as the highest dilution at which the absorbance at 450 nm was at least twice the absorbance of equally diluted pre-immunisation serum samples from the same patient. To obtain the titres, we first fitted the absorbance values of serially diluted serum samples into curves using a four-parameter logistic non-linear regression model in the R programming environment (R Development Core Team 2017, Vienna, Austria). We measured the titre as the dilution at which the curve of the post-immunisation serum crossed the twice multiplied curve of the corresponding pre-immunisation serum. For the purpose of assay consistency and quality, we used quality control samples with two concentrations of the humanised version of mAb DC8E8 (AX004) that was used in the design of AADvac1 [19, 20] as positive controls. AX004 is the passive vaccine counterpart to AADvac1.

### Immunohistochemistry

Tissue samples from AD, Pick’s disease, corticobasal degeneration (CBD) and progressive supranuclear palsy (PSP) were obtained from the Netherlands Brain Bank. Sections were pre-treated with formic acid (98% for 1 min at 4 °C) and heat (autoclave, 121 °C, 20 min), followed by incubation with serum antibodies for 72 h. All sections were incubated with goat anti-human biotinylated secondary antibodies at room temperature for 2 h, and with avidin-biotin-peroxidase complex for 2 h. The immunoreaction was visualised with VIP substrate (VECTASTAIN Elite ABC Kit; Vector Laboratories, Burlingame, CA, USA) and counterstained with methyl green (Vector Laboratories).

### Western blot analysis

We dissolved sarkosyl-insoluble tau [28] isolated from human brain samples (AD: Kuopio Brain Bank, Braak V–VI, gyrus temporalis; PSP and CBD: London Brain Bank, nucleus caudatus and globus pallidus; four AD brains and three different brains for each tauopathy), in 1× SDS sample loading buffer in 1:50 volume of the soluble fraction. We denatured sarkosyl-insoluble tau by heating at 95 °C for 5 min. Samples were loaded onto 5–20% gradient SDS-PAGE gels and electrophoresed in a Tris-glycine-SDS buffer system for 40 min at 25 mA. We transferred the proteins to polyvinylidene fluoride transfer membranes (1 h at 150 mA in 10 mmol/L 3[cyclohexylamino]propanesulphonic acid, pH 12). After the transfer, we blocked the membranes in 5% non-fat dry milk in PBS for 1 h at room temperature and then incubated them for 16 h at 4 °C with serum antibodies diluted in PBS supplemented with 5% bovine serum albumin, followed by three washes with a large volume of PBS supplemented with 0.1% Tween 20 (PBS-T; Sigma-Aldrich, St. Louis, MO, USA). After the washes, we used horseradish peroxidase-conjugated goat anti-mouse Ig (DAKO, Glostrup, Denmark) diluted 1:4000 with PBS-T as the secondary antibody. Incubation (1 h at room temperature) was followed by washing with PBS-T. We developed the blots with SuperSignal West Pico Chemiluminescent Substrate (Pierce Biotechnology) and detected the signal using an LAS3000 imaging system (FUJI Photo Film, Tokyo, Japan).

### Haematology and flow cytometry

Flow cytometry was assessed from the peripheral blood collected into ethylenediaminetetraacetic acid-treated test tubes (VACUETTE 454036; Greiner Bio-One). Samples were analysed within 24 h of blood collection and transported in a temperature-controlled environment (25 °C). For T-cell identification, 50 μl of whole blood was labelled for 20 min in the dark with the following commercial mAbs: CD3 (peridinin chlorophyll protein complex-conjugated, clone UCHT1), CD4 (allophycocyanin [APC]-conjugated, clone MEM-241), CD8 (APC-conjugated, clone MEM-31), CD28 (phycoerythrin-conjugated, clone CD28.2), CD45RA (fluorescein isothiocyanate [FITC]-conjugated, clone MEM-56), CD45RO (FITC-conjugated, clone UCHL1). All antibodies were purchased from Exbio (Prague, Czech Republic). Red blood cells were lysed for 10 min in 100 μl of EXCELLYSE I lysing solution (Exbio). The remaining cells were washed in 2 ml of distilled water, centrifuged for 5 min at 300 *g* and resuspended in 500 μl of PBS for analysis. Finally, samples were acquired on a FACSCalibur flow cytometer (BD Biosciences, Heidelberg, Germany) and analysed with Cell-Quest software (BD Biosciences). Cells were electronically gated according to size (forward scatter) and granularity (side scatter) for the detection of 50,000 leucocytes. For each antibody, negative staining levels were set by comparison to unstained and fluorescence minus one controls. The following lymphocyte populations were analysed: CD3^+^ (T cells), CD3^+^/CD4^+^ (CD4 helper T cells), CD3^+^/CD8^+^ (CD8 cytotoxic T cells), CD3^+^/CD8^+^/CD28^+^ (co-stimulatory receptor CD8 cytotoxic T cells), CD3^+^/CD8^+^/CD28^+^/CD45RA^+^ (naïve co-stimulatory receptor cytotoxic CD8 T cells), CD3^+^/CD8^+^/CD28^+^/CD45RO^+^ (memory co-stimulatory receptor cytotoxic CD8 T cells). Results were expressed as number of the cells with the given phenotype counted per 50,000 leucocytes. Standard haematological assessments were done on a Siemens Advia 2120i haematology system (Siemens Healthcare, Erlangen, Germany) with peroxidase staining technology.

### Cognitive assessments

Cognition was assessed using a standard 11-item Alzheimer’s Disease Assessment Scale cognitive assessment (ADAS-Cog), the Controlled Oral Word Association Test (COWAT) and the Category Fluency Test (CFT). The ADAS-Cog11 is a weighted, multi-domain battery for rating the severity of the patient’s impairment from 0 to 70, with 0 indicating flawless performance and 70 representing pronounced dementia. The MMSE is a short cognitive battery rating the severity of the patient’s impairment from 30 to 0, with 30 indicating flawless performance and 0 representing pronounced dementia. The CFT is an executive function and language test, asking the patient to produce as many words fitting a specific category as possible within 60 s. The category “animals” was used in this study. The COWAT is also a test of language, fluency and executive function. The patient performs three trials, each time being asked to produce as many words as possible starting with a given letter within the allotted timeframe of 60 s per trial. The letters used in the German COWAT version were B, L and S.

### MRI (volumetry)

We used the following scanners: Siemens Magnetom Essenza (1.5 Tesla), Siemens Magnetom Prisma (3 Tesla) and Siemens Magnetom Tim Trio (3 Tesla). MRI was done for safety assessment and volumetric analysis. Scans obtained at screening and in weeks 24, 48, 72 and 96 of treatment were used for the volumetric analysis. The following sequences were recorded: axial T2-weighted (fast spin echo), axial fluid-attenuated inversion recovery (IRfast spin echo), axial T2-star (spoiled gradient echo), and sagittal 3DT1 (IR-prepped fast three-dimensional gradient echo [spoiled]). Detailed scanner settings are listed in Additional file 1. The following ROIs were subjected to volumetric analysis: total brain, hippocampus (sum of left and right) and lateral ventricles.

Whole-brain volume change between follow-up visits and baseline was estimated with SIENA [29], part of FSL [30]. To extract volume estimates of the hippocampus and the lateral ventricles, images were automatically processed with the longitudinal stream [31] in FreeSurfer (version 6.0). Specifically, an unbiased within-subject template space and image was created using robust, inverse consistent registration [32]. Several processing steps, such as skull stripping, Talairach transforms, atlas registration as well as spherical surface maps and parcellations were then initialised with common information from the within-subject template, significantly increasing reliability and statistical power [31].

### Statistical analysis

No sample size calculation was performed, because the study enrolled only patients who completed the preceding study (AxonCO18700). We performed the statistical analyses using Prism version 6.07 software (GraphPad software, La Jolla, CA, USA) and the R programming environment (R Development Core Team 2017). Patients whose signal was at least double compared with baseline in the ELISA for IgG against Axon Peptide 108 at any point of treatment were defined as responders. For correlations between antibody response and other outcomes, all completer patients in whom the dependent variable was recorded at the end-of-study visit were used. For descriptive statistics of antibody titres, responders who completed the study were analysed.

Because the titres of AADvac1-induced IgG antibodies fluctuated over the course of the study depending on whether the patients were recently (re)vaccinated, AUCs were calculated using IgG titre values measured over 96 weeks since the initiation of treatment as a measure of cumulative exposure to IgG antibodies against Axon Peptide 108 (and by proxy against pathological tau protein).

Because patients did not attend the end-of-study visit precisely on the last day of week 96, cubic spline smoothing with generalised cross-validation was used to estimate (if week 96 was between last two consecutive visits) or predict (if week 96 was after last two consecutive visits) values at week 96.

An AUC value corrected for AD severity was also calculated by dividing the IgG titre AUC by the pre-treatment baseline ADAS-Cog11 score. Cognitive outcomes were also analysed in an AD biomarker-positive subgroup (*n* = 10). Biomarker positivity for the subgroup analysis was defined as either medial temporal lobe atrophy rated as 2 or higher on the Scheltens scale, or (in patients who donated cerebrospinal fluid [CSF]) an AD biomarker profile (total tau protein > 400 pg/ml and pT181 tau protein > 60 pg/ml and Aβ42 < 600 pg/ml and Aβ42/Aβ40 ratio < 0.089). The CSF assessment superseded the MRI if the results were contradictory.

Correlations were analysed using Spearman’s correlation coefficient because multiple variables did not follow a normal distribution. To analyse the linear time trend for the variables with lower variability (CD markers), a mixed effects linear regression model was used. For demographic variables that were not re-recorded for this follow-up study (e.g., ApoE4 genotype), the results obtained in the preceding phase 1 trial are imputed. Descriptive statistics are listed as mean (±SD), except for antibody titres, which are listed as geometric means (95% CI), and counts are listed as number of events and percentage of the study population.

### Animal experiments

Transgenic R3/m4 mice (*n* = 10, female, aged ~ 3 months) expressing human truncated tau 151–391/4R under the Thy1 promoter [33] were housed under standard laboratory conditions, with a 12-h/12-h light/dark cycle, and access to food and water *ad libitum*. Animals were treated with five doses of AADvac1 (40 μg of Axon Peptide 108 coupled to KLH) in 21-day intervals.

One animal died spontaneously prior to evaluation. This animal was removed from analysis. Animals were perfused with PBS either at the terminal stage or at the age of 6.5–7 months. Transgenic mice were deeply anaesthetised with Zoletil (Virbac, Carros, France)/xylazine and perfused intracardially using a peristaltic pump for 2 min with PBS. The brain was post-fixed overnight in 4% paraformaldehyde in PBS, pH 7.2, cryoprotected with 15% and 25% sucrose solutions (subsequently overnight), frozen in 2-methylbutane (30 s at − 42 °C) and transferred to dry ice and finally stored at − 70 °C.

Blood samples were obtained at the time mice were killed, and serum was stored at − 70 °C. Antibody half-maximal effective concentration (EC_50_) was measured by ELISA, using tau 151–391/4R as solid phase, and a horseradish peroxidase-conjugated anti-mouse-Ig secondary antibody. We measured the amounts of bound secondary antibodies through the activity of horseradish peroxidase with the ready-to-use chromogenic substrate TMB One (KEM-EN-TEC Diagnostic) and the absorbance at 450 nm. EC_50_ was defined as the dilution at which the sample displays half-maximum absorbance at 450 nm.

Sagittal brainstem sections (40 μm thick) were cut on a Leica CM1850 cryomicrotome (Leica Biosystems, Buffalo Grove, IL, USA). For semi-quantitative analysis, neurofibrillary tangles (NFTs) were quantified in two sections from each brain. Tissue sections were incubated with primary antibodies AT8 (Pierce Endogen), pT212 and pS214 (Invitrogen, Carlsbad, CA, USA) overnight at 4 °C. Sections were immunostained using the standard avidin-biotin-peroxidase method (VECTASTAIN Elite ABC Kit) with VIP as the chromogen.

## Results

The study was done between 18 March 2014 and 10 August 2016. All 28 patients completing the preceding trial were approached for participation in the follow-up study; 26 consented to participate in the follow-up. Four patients discontinued owing to serious adverse events (SAEs), one for an adverse event and one for non-compliance.

Among the 20 patients who completed the final visit, one patient was excluded from cognitive analyses and MRI volumetry, because both the MRI scan and the cognitive profile indicated that this patient had frontotemporal dementia (FTD) and not AD. One patient was not able to complete the ADAS-Cog11 at the final visit; two patients were not able to complete the COWAT and CFT; low image quality prevented the volumetric analysis in one completer.

### Immunogenicity

**Fig. 1:**
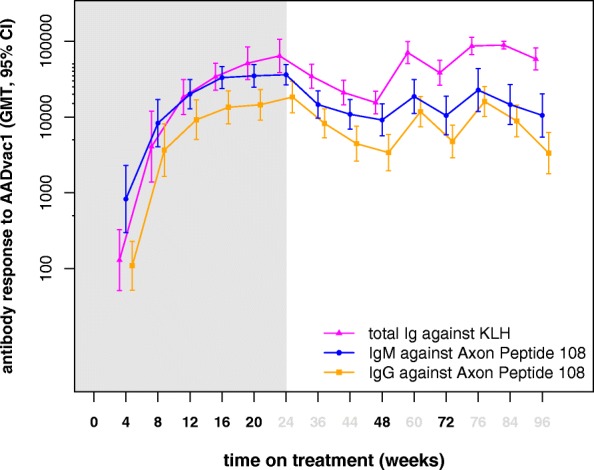
Antibody response to AADvac1 over 96 weeks of treatment. Values shown indicate geometric mean titres and 95% CI of the geometric mean. Time points of AADvac1 administration indicated by bold treatment weeks. Light grey area indicates values obtained in the first-in-human study

Antibody titres increased following each of the six doses in the initial vaccination regimen. Over the 6-month vaccination-free period thereafter, antibody titres declined in a majority of patients: IgG to a median value of 15.8%, IgM to 23.5%, and anti-KLH antibodies to 19.8% of values obtained 4 weeks after the initial vaccination regimen. Booster doses restored all types of antibody titres to previously achieved levels (*see* Fig. 1 and Table 2).

**Table 2:** Antibody titres over 96 weeks of AADvac1 treatment

	GMT													
	Week + 4	Week + 8	Week + 12	Week + 16	Week + 20	Week + 24	Week + 36	Week + 44	Week + 48	Week + 60	Week + 72	Week + 76	Week + 84	Week + 96
Anti-KLH	**260.0**	**8128**	**36,784**	**68,023**	**102,453**	**127,972**	68,870	42,043	30,967	141,323	76,711	172,828	177,672	116,545
(lower CI)	**103.4**	**2763**	**21,622**	**45,268**	**62,490**	**77,347**	47,757	28,870	21,922	101,408	52,550	132,513	158,315	83,702
(upper CI)	**653.7**	**23,906**	**62,578**	**102,216**	**167,973**	**211,734**	99,316	61,225	43,742	196,949	111,982	225,409	199,395	162,275
Anti-peptide IgG	**219.1**	**7318**	**18,382**	**26,868**	**29,047**	**36,697**	16,483	8908	6789	23,541	9541	32,023	17,741	6679
(lower CI)	**104.5**	**3300**	**10,084**	**16,320**	**18,244**	**22,759**	10,642	5233	3913	14,811	5819	20,397	10,941	3580
(upper CI)	**459.2**	**16,228**	**33,509**	**44,233**	**46,246**	**59,171**	25,529	15,163	11,781	37,417	15,646	50,273	28,767	12,462
Anti-peptide IgM	**1659**	**16,586**	**40,048**	**66,087**	**69,789**	**72,154**	29,150	21,702	18,272	37,301	20,904	45,288	29,153	21,018
(lower CI)	**596.7**	**8075**	**25,606**	**47,284**	**49,429**	**53,114**	19,298	13,862	11,250	22,220	11,621	23,581	15,903	10,887
(upper CI)	**4612**	**34,068**	**62,634**	**92,366**	**98,536**	**98,019**	44,032	33,976	29,676	62,616	37,602	86,980	53,443	40,575

*Ig* Immunoglobulin, *KLH* Keyhole limpet haemocyanin

Basic vaccination regimen is shown in bold

Similarly to the antibody response observed in the first-in-human study, the 96-week AUC of the IgG antibody response was varied, with a median AUC of 1.60 × 10^6^ (IQR, 0.96 × 10^6^–3.93 × 10^6^; minimum, 0; maximum, 7.4 × 10^6^). Patients who initially responded to AADvac1 treatment with high antibody titres continued to display high levels of antibody response; their AUC of IgG titre was highly correlated with the IgG titre at the end of the primary vaccination regimen (Spearman *r* = 0.889, *p* < 0.001). Over the entire course of treatment, IgG antibody titre against the peptide was correlated to anti-KLH titres (Spearman *r* = 0.595, *p* < 0.001).

A portion of the immunological predictors identified in the first-in-human study also predicted the cumulative amount of antibody production over 96 weeks. The IgG AUC values correlated with the following immunological variables measured prior to vaccination: absolute (*r* = 0.618, *p* = 0.004) and relative lymphocyte counts (*r* = 0.755, *p* = 0.001), CD3^+^/CD4^+^ lymphocyte counts (*r* = 0.585, *p* = 0.007), as well as absolute (*r* = − 0.492, *p* = 0.028) and relative segmented neutrophil granulocyte counts (*r* = − 0.786, *p* < 0.001). A negative correlation between age and IgG AUC was observed (*r* = − 0.480, *p* = 0.032). Relative lymphocyte and segmented granulocyte counts show a far stronger association with age, though (*r* = − 0.667, *p* = 0.001 and *r* = 0.642, *p* = 0.002, respectively). Similarly, there was a negative correlation between age and CD3^+^/CD4^+^ lymphocyte counts prior to treatment (*r* = − 0.485, *p* = 0.009).

No significant impact of sex on IgG AUC was observed; as expected, an impact of sex on several haematological variables was noticeable, though (*p* < 0.05 for CD3^+^, CD3^+^/CD4^+^, CD3^+^/CD8^+^/CD28^+^, CD3^+^/CD8^+^/CD28^+^/CD45RA^+^, and relative lymphocyte counts, as well as relative and absolute segmented neutrophil granulocyte counts).

No changes in lymphocyte populations were observed over the course of treatment.

**Fig. 2:**
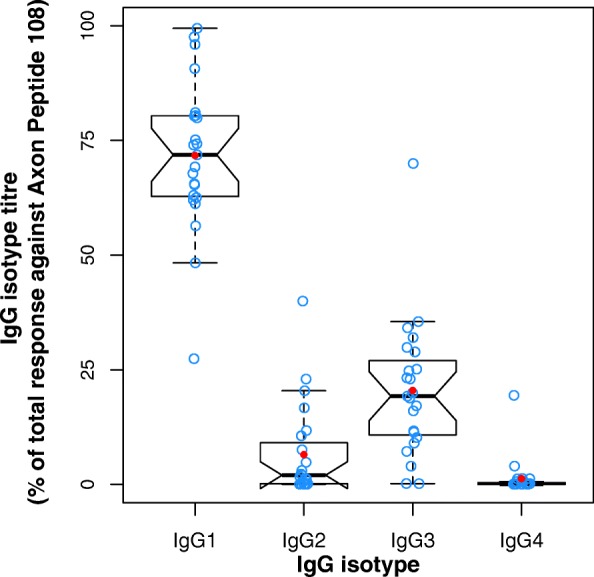
The antibody response to AADvac1 is immunoglobulin G1-dominated. Values were obtained after the sixth dose of AADvac1

The IgG subclass assessment was performed cross-sectionally at the end of the initial vaccination regimen. In all patients who mounted an IgG response, the response was IgG1-dominated: the patients’ IgG response consisted of 69.54% (61.85, 78.20) IgG1, 1.33% (0.50, 3.50) IgG2, 12.58% (6.70, 23.63) IgG3, and 0.17% (0.08, 0.39) IgG4 (geometric mean, 95% CI) (*see* Fig. 2).

**Fig. 3:**
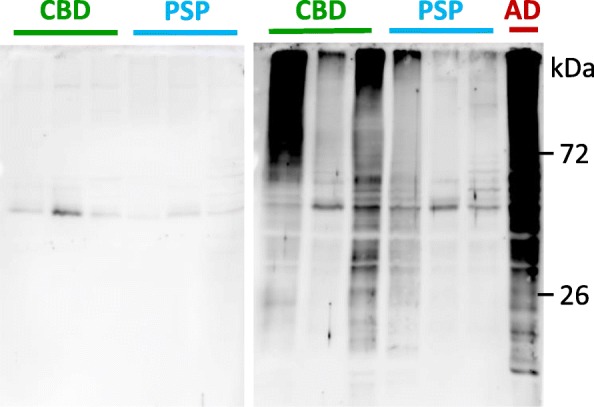
AADvac1-induced antibodies label tau extracts from corticobasal degeneration (CBD) and progressive supranuclear palsy (PSP) brains. Left: Patient R17, pre-treatment serum. Right: Serum of the same patient after six doses of AADvac1. The serum labels both high-molecular-weight aggregates and low-molecular-weight fragments of tau protein. Staining of Alzheimer’s disease (AD) brain extract is shown as a positive control

Western blot analysis was used to assess whether the antibodies induced by vaccination with AADvac1 could label pathological tau protein in brains with AD and non-AD tauopathies (CBD, PSP). AADvac1-induced antibodies are reactive with sarkosyl-insoluble tau extracts in AD as well as non-AD tauopathies (three PSP and three CBD brains) (Fig. 3).

**Fig. 4:**
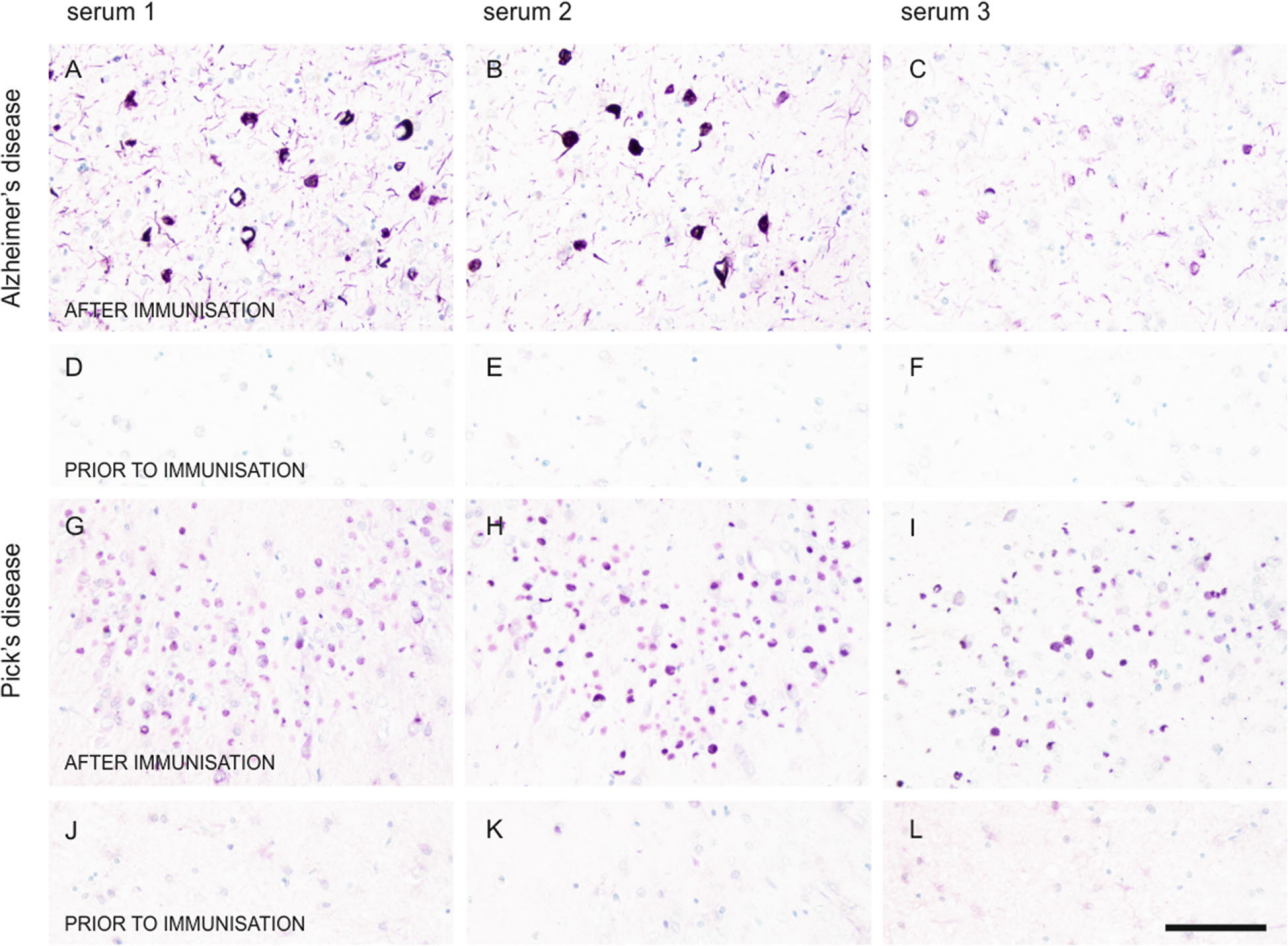
Sera of patients treated with AADvac1 recognise tau pathology in Alzheimer’s disease and Pick’s disease. **a**–**f** Alzheimer’s disease. **g**–**l**: Pick’s disease. Sera of three different patients with different strengths of antibody responses (patient R17 with an anti-AD-tau titre of 1:30,999; patient R25 with 1:18,185; and patient R10 with 1:12,800) were used for staining. Staining with pre-treatment sera is shown as a negative control (**d**–**f**, **j**–**l**)

**Fig. 5:**
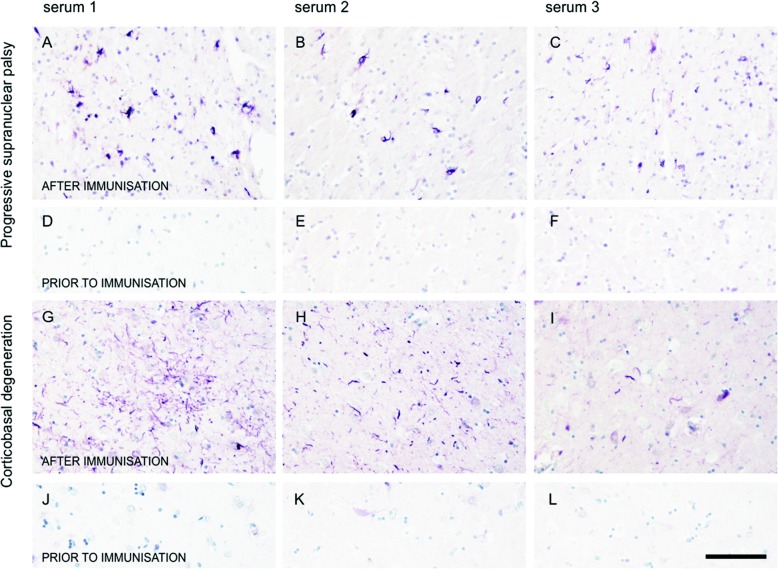
Sera of patients treated with AADvac1 recognise tau pathology in progressive supranuclear palsy (PSP) and corticobasal degeneration (CBD). **a**–**d** PSP. **g**–**l** CBD. Sera of three different patients with different strengths of antibody response (patient R17 with an anti-AD-tau titre of 1:30,999; patient R25 with 1:18,185; and patient R10 with 1:12,800) were used for staining. Staining with pre-treatment sera is shown as a negative control (**d**–**f**, **j**–**l**)

Antibodies elicited by vaccination were able to label all aspects of tau pathology in histological preparations from AD brains: NFTs, neuropil threads, and the neuritic corona of plaques (Fig. 4a–c). Similarly, the same patients’ sera were able to stain the Pick bodies in Pick’s disease (Fig. 4g–i), oligodendroglial coiled bodies in PSP (Fig. 5a–c) and astrocytic plaques in CBD (Fig. 5g–i).

Antibodies against Axon Peptide 108 were detected in the CSF of all patients who donated it. The titres in the CSF were 0.22 (±0.13)% of those measured in the periphery (mean ± SD) and strongly correlated (Pearson *r* = 0.884, *p* = 0.0003).

### Safety

The only adverse events clearly linked to AADvac1 treatment were injection site reactions (erythema, swelling, warmth, pruritus, pain, nodule); these are usual for aluminium-containing vaccines. One or more of these were observed in 50% of patients on AADvac1 treatment. Injection site reactions were reversible and predominantly mild in presentation.

Six serious adverse events were observed (abdominal strangulated hernia, dehydration, acute psychosis, behavioural and psychiatric symptoms of dementia, second-degree atrioventricular block, and sinus bradycardia). None were judged by the investigators to be related to AADvac1 treatment.

No allergic or anaphylactic reactions were observed. No safety signals emerged in laboratory assessment (coagulation, blood biochemistry, haematology, and urinalysis), vital sign assessment, or neurological and physical examination. No safety signals were detected by MRI assessment. No oedematous changes occurred; no meningeal changes and no meningoencephalitis were observed. New micro-haemorrhages were observed in one ApoE4 homozygote, and superficial haemosiderin was detected in one ApoE4 heterozygote; both events were clinically silent. This is consistent with the background incidence of such lesions in the AD patient population [34].

All adverse events occurring in at least 10% of patients are listed in Table 3.

**Table 3:** Adverse events observed in at least 10% of patients in the AC-AD-002 study, by system organ class and preferred term

System organ class	Overall		
	(*N* = 26)		
Preferred term	No.	(%)	Events
General disorders and administration site conditions			
Injection site erythema	8	30.8	14
Injection site swelling	7	26.9	10
Injection site warmth	4	15.4	4
Injection site pruritus	3	11.5	4
Nervous system disorders			
Cerebral atrophy	5	19.2	5
Restlessness	3	11.5	3
Metabolism and nutrition disorders			
Abnormal weight gain	3	11.5	3
Psychiatric disorders			
Depression	3	11.5	4
Behavioural and psychiatric symptoms of dementia	3	11.5	3
Infections and infestations			
(Genito)urinary tract infection	4	15.4	4

*N* = number of patients

*n* = number of patients who experienced the AE

% = percentage of patients who experienced the AE

The term ‘Behavioural and psychiatric symptoms of dementia’ includes one instance of ‘Aggression’, one instance of ‘Acute psychosis’ and one instance of unspecified ‘Behavioural and psychiatric symptoms of dementia’

### Cognition

Over 96 weeks of observation, on average, patients declined on the ADAS-Cog11, the COWAT, the CFT, and the MMSE. The mean change (±SD) on the ADAS-Cog11 was 11.7 (±13.0) points, on the COWAT -4.7 (±7.3), on the CFT –2.2 (±4.6), and on the MMSE –5.3 (±6.6). Split by disease stage (mild AD defined as MMSE 20–26 and moderate AD as MMSE 15–19 at screening), the change in the mild AD population on the ADAS-Cog11 was 7.6 (±11.2), on the COWAT –2.9 (±8.5), on the CFT –1.4 (±5.6), and on the MMSE –3.2 (±7.2); the change in the moderate AD population on the ADAS-Cog11 was 18.1 (±13.9), on the COWAT –7.3 (±4.2), on the CFT –3.4 (±2.6), and on the MMSE –8.3 (±4.8).

**Fig. 6:**
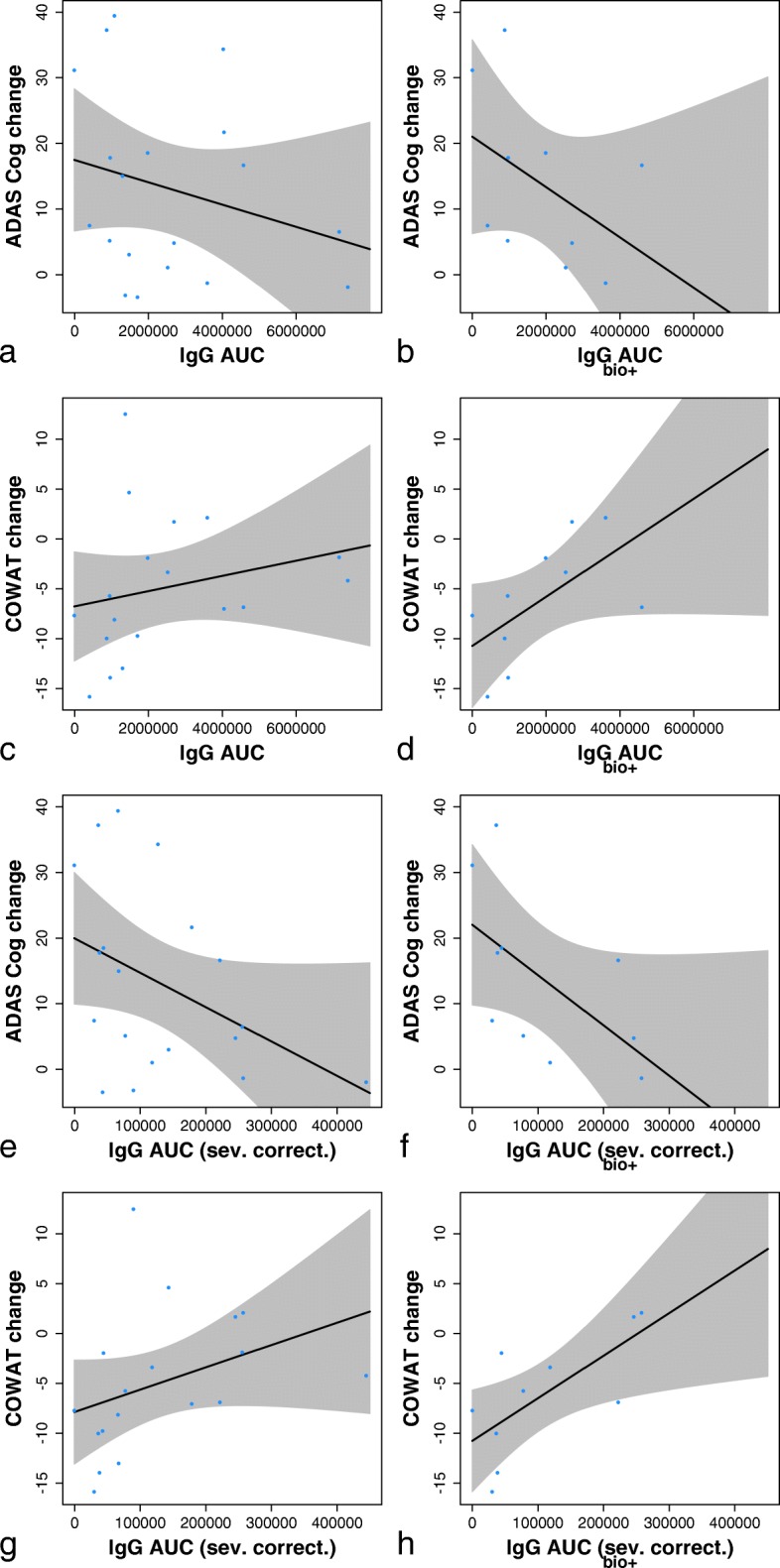
Change in cognition (Alzheimer‘s Disease Assessment Scale 11-item cognitive assessment [ADAS-Cog11], Controlled Oral Word Association Test [COWAT]) over 96 weeks, displayed as correlation with the immunoglobulin G (IgG) titre AUC. Results are shown for completers (left) and for patients with a positive biomarker profile (right). Results are shown with raw AUC values (**a**–**d**) and with AUC values corrected for disease severity (**e**–**h**). Category Fluency Test results were inconclusive (not shown)

Cognitive change measured by the COWAT correlated with IgG titre AUC, with patients who had higher IgG titres experiencing less cognitive decline (Spearman *r* = 0.488, *p* = 0.049) (Fig. 6c).

As is usual with AD populations recruited solely on the basis of clinical criteria, some patients were negative for AD biomarkers (hippocampal atrophy on MRI, and/or CSF AD biomarkers). Therefore, we analysed the correlation of cognitive change with IgG AUC also in the subgroup of patients with positive AD biomarkers according to McKhann [35]. A clearer association between cognitive benefit and IgG AUC was seen for COWAT (Spearman *r* = 0.673, *p* = 0.039); the association for ADAS-Cog11 likewise was stronger, but did not reach statistical significance (Spearman *r* = − 0.576, *p* = 0.088) (Fig. 6b, d).

Finally, we tested the hypothesis whether patients with more advanced disease, and thus presumably more tau pathology, require higher antibody titres for cognitive benefit. The IgG AUCs were divided by the respective patient’s baseline ADAS-Cog11 value, and analysed for association with cognitive change. Correcting thus for baseline disease severity, the association between IgG AUC and cognitive change was significant both for the ADAS-Cog11 (Spearman *r* = − 0.758, *p* = 0.015) and the COWAT (Spearman *r* = 0.806, *p* = 0.007) (Fig. 6f, h).

The change in CFT was correlated with IgG AUCs only in the general population; the association was not found in the subgroup analysis or when the IgG titre AUCs were corrected for disease severity.

### MRI volumetry

The changes in brain volume observed over 96 weeks of treatment, shown as mean (SD), were as follows: TBV –5.433% (2.448); LVV + 16.84% (8.946); HCV –9.324% (3.488). These atrophy rates do not exceed those expected of mild to moderate AD populations [36, 37].

**Fig. 7:**
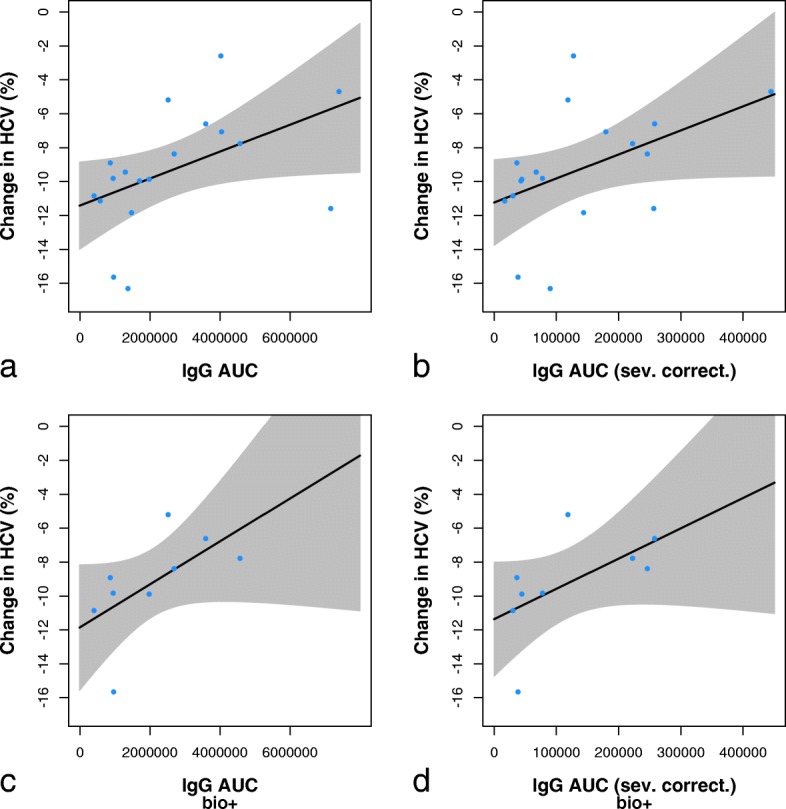
Hippocampal atrophy over 96 weeks, displayed as correlation with the immunoglobulin G (IgG) titre AUC. Results are shown for completers (**a**, **b**) (*n* = 18) and for patients with a positive biomarker profile (**c**, **d**) (*n* = 9). Results are also shown with raw AUC values (**a**, **c**) and with AUC values corrected for disease severity (**b**, **d**). One patient with frontotemporal dementia and one patient with poor magnetic resonance imaging scan quality were excluded. *HCV* Hippocampal volume

Analogously to cognitive assessment, correlations between AUC of IgG titres and brain atrophy were assessed for all completers and for the biomarker-positive subgroup. In the overall population, high IgG titre AUCs (both uncorrected and corrected for disease severity) were associated with less hippocampal atrophy (*r* = 0.544, *p* = 0.0196 and *r* = 0.476, *p* = 0.0460); no association was found for whole-brain volume and ventricular volume. In the biomarker-positive subgroup, a similar relationship between high IgG antibody titres and low hippocampal atrophy was observed (*r* = 0.683, *p* = 0.050 for uncorrected, and *r* = 0.750, *p* = 0.025 for corrected titres); no significant correlation was seen for total brain and ventricular volume (Fig. 7).

### Transgenic animals: correlation of antibody response and NFT counts

To evaluate whether the biological response in the central nervous system (CNS) is proportional to the strength of the AADvac1-induced antibody response, we administered AADvac1 to R3/m4 transgenic mice. These animals develop progressive and ultimately lethal neurofibrillary degeneration. Their antibody response was evaluated (mean EC_50_, 8088; minimum, 4639; maximum, 12,704), and compared with the number of NFTs in the midbrain. Animals with stronger antibody response had consistently lower tangle counts than those with weak antibody response. The correlation was significant for all three phospho-antibodies (AT8, anti-pS214, anti-pT212) used for evaluation of neurofibrillary pathology (AT8, *r* = − 0.860, *p* = 0.006; pS214, *r* = − 0.747, *p* = 0.033; pT212, *r* = − 0.921, *p* = 0.003). *See* Additional file 1 for figures.

## Discussion

Previously, we have reported data from the 24-week first-in-human study of AADvac1 [17]. To highlight the salient points, 29 of 30 patients have developed an IgG response against the tau peptide component of AADvac1; patients were also shown to mount an immune response against recombinant pathological tau 151–391/4R, and against sarkosyl-insoluble tau extracts from AD brains. The antibodies showed a pronounced preference for AD tau over healthy tau protein. The safety profile in the preceding study was beneficial, with injection site reaction being the only adverse event clearly tied to AADvac1 treatment, showing that it is possible to safely vaccinate against pathological tau protein. The exploratory analysis of cognitive status showed patients being (on average) stable in cognition over 24 weeks. The topic of the present article is the follow-up study of said trial, and data over further 72 weeks are illustrated next to the first-in-human study data to provide context.

Tau pathology is composed of a wide range of aberrant tau protein forms. These come into being through post-translational modification of the native protein. Of these, truncation and hyperphosphorylation appear to be key for the transition of physiological tau protein into its diseased state [38, 39]. On one hand, the possible pathological forms of tau are legion, because the combinations of reported phosphorylation patterns and truncation sites are truly countless. On the other hand, the profound changes that tau undergoes in disease create features that can be immunologically targeted: hyperphosphorylation leads to the appearance of novel phospho-epitopes; truncation leads to loss of epitopes on the N- and/or C-terminus of the tau molecule, but creates novel conformational epitopes on the remainder of the tau protein through a global conformational change of the molecule, and new truncation-dependent epitopes at the new termini [40, 41]. An ideal immunotherapeutic would be able to capture the entire range of pathological tau forms while being sufficiently specific to fish them out from between healthy tau molecules.

Basically, all parts of the tau molecule have been targeted by immunotherapies in preclinical studies; curiously, only immunotherapies against the N-terminus (C2N 8E12, RO 7105705, and BMS-986168), against the MTBR (AADvac1), and against the molecule’s C-terminus (ACI-35) were moved forward into clinical development [13]. All tau forms capable of seeding and aggregation contain the MTBR, and it is via this region that the protein aggregates [42], with other parts of the molecule extending outward to form a fuzzy coat or breaking off, leading to truncation. Thus, not all pathological tau species need to contain the N-terminus, and in fact they often do not [43]. The strategies behind the aforementioned compounds fundamentally differ. The therapies focusing on the N-terminus target epitopes that are not specific to diseased tau, seeking to prevent the spreading of tau, and possibly also to reduce the level of physiological tau. The risks here are twofold: Many tauons that do not have the N-terminus escape the attention of these compounds and continue spreading; furthermore, reduction of healthy tau can be detrimental [44]. Most current immunotherapy approaches targeting extracellular tau do not distinguish between pathogenic forms of tau that are thought to propagate disease and the forms of extracellular tau that are found in the healthy brain [45]. Immunotherapies targeted at the MTBR have the potential to target all aggregating tau forms and tauons, whether truncated or not, and owing to the conformational change of this region in misfolded tau, discrimination between healthy and diseases tau is feasible [17, 20].

We have previously reported the identification of a conformational epitope that is functionally important for tau protein aggregation, is conserved throughout the manifold manifestations of tau pathology in AD, and allows the immunological differentiation between healthy and diseased tau [20]; this epitope is the target of AADvac1. In line with the above theory, the induced antibodies were able to label both brain extracts and histological preparations from AD and non-AD tauopathies, indicating that the conformational epitope in the microtubule-binding region of tau that is targeted by AADvac1 is both present and accessible in all evaluated forms of tau pathology (AD, PSP, CBD, Pick’s disease). This finding aligns with the recently described cryogenic electron microscopic structures of tau filaments [42], which show that the DC8E8 binding motif [20] is accessible even in the microtubule-binding region that forms the filament core of AD tau. Because the microtubule-binding region is absolutely necessary for all forms of tau aggregation [46, 47] and thus a common trait of all pathological tau forms, AADvac1 has the potential to be a disease-modifying therapy in non-AD tauopathies as well. Based on the fact that the antibody response was dominated by IgG1 and, to a lesser extent by IgG3, the antibodies elicited by AADvac1 vaccination could engage microglia in the removal of pathological tau protein via their effector functions.

We have previously shown that antibody titres following the primary vaccination regimen correlate with several immunological variables [17]; the cumulative amount of IgG antibodies produced by patients over 96 weeks, expressed by the IgG titre AUC value, was similarly tied to pre-treatment relative and absolute lymphocyte and segmented granulocyte counts, and relative CD3^+^/CD4^+^ lymphocyte counts, with segmented granulocytes having a profound negative impact on IgG titre. Lymphocyte populations were reported to decline with age [48], and a similar behaviour was seen in this study, with higher age having a negative impact on lymphocyte counts, and consequently on IgG titres as well. The connection between titres and immunological values is far closer than between titres and age, indicating that in the elderly, immunological health matters more than listed age in regard to immune response to novel antigens. It is clear that immunological health should be analysed in vaccine trials in the elderly to better understand the trial populations and to be able to explain the variability in immune response.

Owing to differences in methodology, it is difficult to quantitatively compare the AADvac1-induced antibody response to that induced by other active immunotherapies in AD (where data are published). With AN1792, the observed antibody titres against the immunogen reached titres of 1:2200 in 19.4% of patients [49], with AADvac1 in 96.4%. Owing to the set-up of the assay used to measure antibody response in the development of CAD106, it is difficult to compare absolute titre values, but the responder rate with the higher 450-μg dose (89.2%) was similar to that seen with AADvac1 [18]. Other fields have benefitted from establishing comparable methodology and reference standards [14]; we firmly believe that similar efforts should be initiated in AD to compare staining patterns in brain extracts and strength of antibody response in active immunotherapies.

The AADvac1-induced IgG antibody response persisted over 6 months without vaccination, though decreased to < 20% of previously achieved values. Re-vaccination restored previous levels of antibodies, and patients with higher initial antibody response continued to display high IgG values throughout the study. Based on these findings, more frequent booster doses are indicated in future studies to maintain higher antibody levels. Antibodies raised by AADvac1 were detected in the CSF of study patients, showing that they can pass into the CNS.

The safety profile of AADvac1 over a total treatment duration of 96 weeks was favourable, with local injection site reactions being the only adverse event clearly linked to treatment. No correlation between the strength of the immune response and adverse events was observed. The observed adverse events were consistent with the expected background incidence in elderly AD populations [34, 50]. This marks AADvac1 as a compound that is suitable for treatment of patients for extended periods of time. This is vital in an indication such as AD: If a compound is shown to be efficacious in halting the progress of AD neurodegeneration, it may possibly need to be administered for decades, especially if advances in other fields further prolong life expectancy.

In the absence of a control group, the observed cognitive changes in study patients are best put into context by contrasting them to the expected cognitive decline in similar populations and by comparing the cognitive change to the titres of AADvac1-induced antibodies. Because the antibody induced by immunisation is the molecule that mediates the effect of a vaccine such as AADvac1, the patients were in essence on different doses of the active molecule (by a factor of ~ 18 between the weakest and strongest responders). The study’s findings indicate that patients who were most likely to have AD declined less if they had higher AADvac1-induced IgG titres, especially so if they had high IgG titres in comparison to the severity of their disease (thus, presumably, a higher antibody-to-pathology ratio), constituting a dose-dependent effect of AADvac1-induced antibodies on cognitive decline. Owing to the limited dataset, it is not possible to calculate an immunological correlate of protection; given the favourable safety profile, the focus in further development should be on raising and maintaining IgG titres as high as possible. As for absolute change, it is perhaps best compared with placebo groups of the recent phase 3 studies of mAbs in mild to moderate AD populations (e.g., bapineuzumab) [51]. Said studies showed a 78-week decline of 8.7 ± 10.4 on the ADAS-Cog11 (mean ± SD; 95% CI of the mean, 7.7–9.7); on AADvac1 treatment, the 72-week decline was 6.0 ± 9.2 points, and the 84-week decline was 7.6 ± 10.1 points (mean ± SD) (95% CI of the mean, 1.4–10.6 and 1.6–15.1, respectively).

The MRI volumetry findings were in line with the above observations. Although overall the total brain volume and hippocampal volume decreased and ventricles expanded as one would expect of an AD population, the hippocampi shrank less in patients with high AADvac1-induced antibody titres.

The animal findings provide a plausible explanation for the above-described effects. We have shown that AADvac1-induced antibodies against pathological tau protein reduced NFT counts in the brains of the mice in a dose-dependent manner. In humans, NFT counts closely correlate with cognitive impairment [2] and brain atrophy [8]; thus, the above-mentioned titre-dependent reduction in cognitive decline is best explained by a reduction of NFT accumulation.

These findings are consistent with what would be observed with a treatment that is efficacious, but they are not conclusive proof of efficacy; they are limited by unblinded analysis, absence of a placebo group, the small sample size of the biomarker-positive subgroup (*n* = 10), and the fact that the subgroup analysis was not pre-specified.

Further studies are necessary to expand the safety database, optimise immunogenicity, and evaluate the efficacy of AADvac1.

## Conclusions

AADvac1 treatment of patients with mild-to-moderate AD was safe. All patients who previously displayed an IgG antibody response to the tau peptide component of AADvac1 retained it, though re-vaccination was indicated. More frequent re-vaccination appears warranted in future studies. As intended, the dominant IgG isotype was IgG1. The antibodies reacted with all evaluated tisssues and brain extracts from AD and non-AD tauopathies, highlighting that the AADvac1-induced antibody response targets a common denominator of all assessed tau pathologies. This indicates a potential for cross-applicability of AADvac1 in non-AD tauopathies. Patients with higher antibody titres had a tendency towards slower brain atrophy and less cognitive decline; these findings are encouraging, but need to be confirmed in larger, placebo-controlled studies.

## Additional file

Additional file 1: Supplementary material for FUNDAMANT: An interventional 72-week phase 1 follow study of AADvac1, an active immunotherapy against tau protein pathology in Alzheimer’s disease. (DOCX 4564 kb)
