# Supplementary material for: FUNDAMANT: an interventional 72-week phase 1 follow-up study of AADvac1, an active immunotherapy against tau protein pathology in Alzheimer’s disease
Source: Alzheimers Res Ther. 2018 Oct 24;10:108. doi: 10.1186/s13195-018-0436-1 (PMC6201586; doi:10.1186/s13195-018-0436-1)
Supplement: Supplementary file 1 — Supplementary material for FUNDAMANT: An interventional 72-week phase 1 follow study of AADvac1, an active immunotherapy against tau protein pathology in Alzheimer’s disease. (DOCX 4564 kb) [file 13195_2018_436_MOESM1_ESM.docx]

Supplementary material

For "FUNDAMANT: An interventional 72-week phase 1 follow study of AADvac1, an active immunotherapy against tau protein pathology in Alzheimer’s disease" (Novak et al., 2018)

Table of Contents

[Abbreviations 1](#_Toc524100504)

[Relationship of the phase 1 first-in-man study and FUNDAMANT follow-up 3](#_Toc524100505)

[Inclusion and exclusion criteria of preceding phase 1 study 4](#_Toc524100506)

[Inclusion criteria (Axon CO18700) 4](#_Toc524100507)

[Exclusion criteria (Axon CO18700) 4](#_Toc524100508)

[Inclusion and exclusion criteria of the AC-AD-002 follow up study 6](#_Toc524100509)

[Inclusion criteria (AC-AD-002) 6](#_Toc524100510)

[Exclusion criteria (AC-AD-002) 6](#_Toc524100511)

[Subject disposition 7](#_Toc524100512)

[Histological comparison of AADvac1-induced antibodies to reference antibodies 8](#_Toc524100513)

[Histology (non-demented control brain) 9](#_Toc524100514)

[Staining of AD brain extracts with AADvac1-induced antibodies 10](#_Toc524100515)

[CSF anti-peptide response vs. serum anti-peptide response 11](#_Toc524100516)

[MRI settings 11](#_Toc524100517)

[Longitudinal change on ADAS-Cog, CFT, LFT, and MMSE 15](#_Toc524100518)

[IgG titre AUC vs. brain atrophy 17](#_Toc524100519)

[Longitudinal brain atrophy 23](#_Toc524100520)

[AADvac1-induced antibody titres vs. NFT counts (mice) 26](#_Toc524100521)

# Abbreviations

Aβ40, Aβ42 = amyloid-β (40- or 42-amino acid form)

AChEI = Acetylcholine esterase inhibitor

AD = Alzheimer's disease

ADAS-Cog11 = Alzheimer's disease assessment scale – cognitive (11-item)

AE = Adverse event

AUC = Area under the curve

CBD = Corticobasal degeneration

CFT = Category fluency test

CI = Confidence interval

CNS = Central nervous system

COWAT = Controlled oral word association test

CSF = Cerebrospinal fluid

DSMB = Data and safety monitoring board

DICOM = ”digital imaging and communication in medicine” standard

EDTA = Ethylenediaminetetraacetic acid

ELISA = Enzyme-linked immunosorbent assay

FLAIR = Fluid attenuated inversion recovery

FMO = Fluorescence-minus-one

FOV = Field of view

FTD = Frontotemporal dementia

GDS = Geriatric Depression Scale

HCV = Hippocampal volume

IgG = Immunoglobulin G

IgM = Immunoglobulin M

IQR = Interquartile range

IR-TFE = Inversion recovery – turbo field echo

KLH = Keyhole limpet haemocyanin

LVV = Lateral ventricle volume

mAbs = Monoclonal antibodies

MMSE = Mini Mental State Examination

MRI = Magnetic Resonance Imaging

MTBR = Microtubule binding repeats

NINCDS-ADRDA = National Institute of Neurological and Communicative Disorders and Stroke and the Alzheimer’s Disease and Related Disorders Association

NEX = Number of excitations (= number of averages)

NFT = Neurofibrillary tangle

PBS = Phosphate buffered saline

PiD = Pick's disease

PSP = Progressive supranuclear palsy

pT181 = phospho-threonine at the amino acid position 181

RF = radio frequency

SAE = Serious adverse event

SD = standard deviation

SPGR = spoiled gradient echo

TBV = Total brain volume

TE = echo time (time of echo)

THK = thickness

TI = inversion time (time of inversion)

TR = repetition time (time of repetition)

3D = three dimensional

# Relationship of the phase 1 first-in-man study and FUNDAMANT follow-up


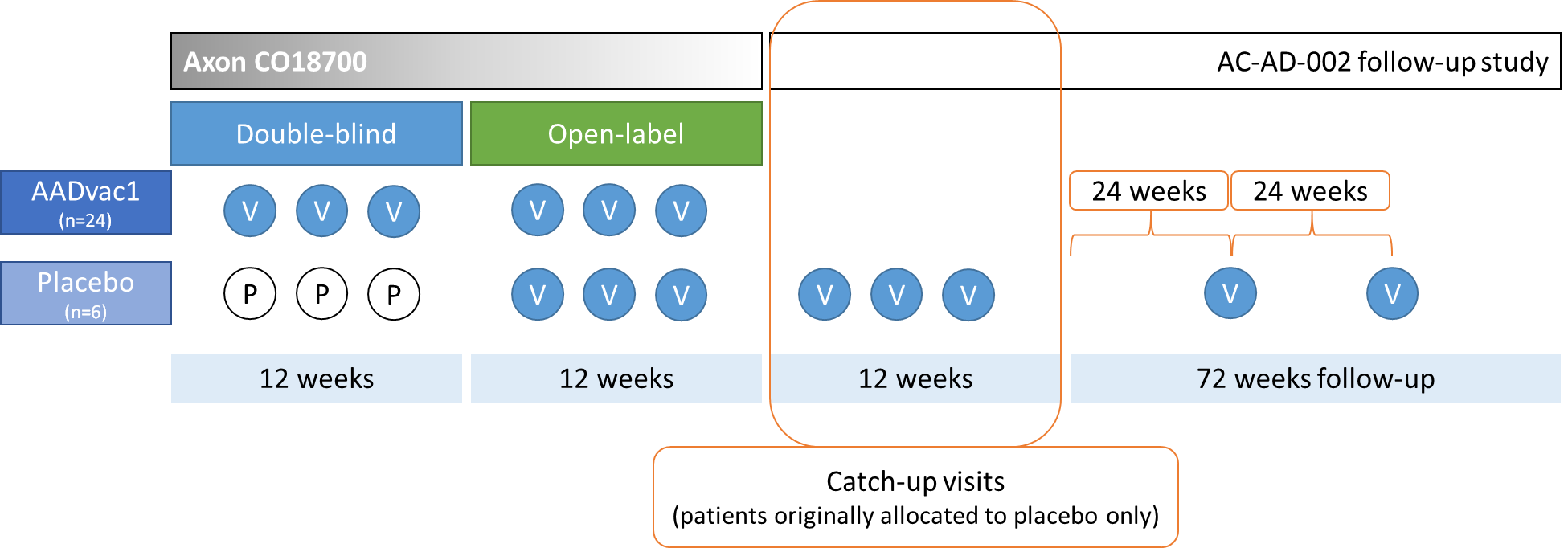


**Figure 1: Alignment of the phase 1 first-in-man study AxonCO18700, and the follow-up FUNDAMANT study**

A subset of patients (n=6) were allocated to placebo in the double-blind phase of the first-in-man study. These patients were later switched to receive AADvac1. To bring their regimen in line with patients allocated to AADvac1 from the study start, placebo patients (and only they) have attended so‑called "catch-up visits", where they received 3 doses of AADvac1. Thus, both groups have received a basic vaccination regimen of 6 doses of AADvac1 in 4-week intervals, followed by 2 boosters in 24‑week intervals (see **Figure 1** and **Figure 2**).


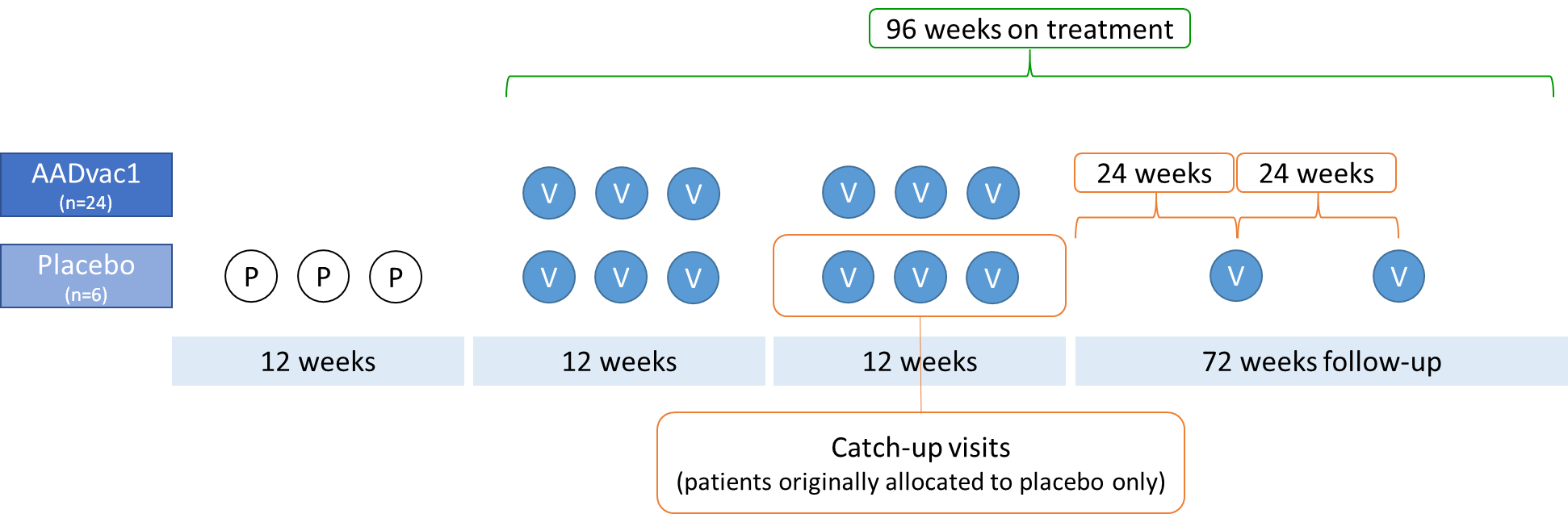


**Figure 2: Both patients initially allocated to AADvac1 and placebo patients were 96 weeks on AADvac1 treatment, with an initial dosing regimen of 6 AADvac1 doses, followed by 2 boosters.**

Most results in this manuscript are presented as "change over 96 months of treatment" and disregard the data gathered on 3 months of placebo treatment. The results gathered immediately prior to the first AADvac1 dose are considered to be the pre-treatment baseline for patients initially allocated to placebo.

# Inclusion and exclusion criteria of preceding phase 1 study

The criteria based on which patients were enrolled in the preceding phase 1 first-in-man study are replicated below.

## Inclusion criteria (Axon CO18700)

The subjects have to meet the following criteria upon enrolment:

1. Diagnosis of probable Alzheimer’s disease based on the NINCDS/ADRDA criteria.
2. Assessing the severity of Alzheimer’s disease of mild to moderate degree by the Mini Mental State Examination (MMSE). AD of mild to moderate degree will be considered confirmed if the MMSE score is in the range of 15 to 26.
3. Being on a stable dose of Alzheimer’s Disease treatment since 3 months before screening visit or being untreated.
4. Hachinski Ischemia Scale is used to distinguish AD from multi-infarct dementia. A score of ≤ 4 suggests AD.
5. The result of the Magnetic Resonance Imaging scan (MRI) of the patient’s brain has to be consistent with the diagnosis of AD.
6. Informed consent capability (as determined by an independent neurologist / psychiatrist).
7. Written informed consent signed and dated by the patient and the caregiver.
8. Age between 50 and 85 years *(inclusive)*.
9. Availability of a partner/caregiver knowing the patient and being able to accompany the patient to the visits.
10. Adequate visual and auditory abilities and German language skills to allow neuropsychological testing.
11. Female patients are only eligible for the study if they are either surgically sterile or at least 2 years postmenopausal.
12. A potential participant has to be on stable doses of all medications he/she is taking because of concomitant illnesses according to medical history for at least 30 days prior to Visit 1 if considered relevant by the investigator.
13. Sexually active males must be using reliable contraception methods (i.e. condoms) or be surgically sterile.

## Exclusion criteria (Axon CO18700)

Subjects presenting with any of the following upon enrolment will not be included into the study:

1. Pregnant or breastfeeding women.
2. Participation in another clinical trial within 3 months before Visit 1.
3. Patients not expected to complete the clinical trial.
4. Presence or history of allergy to components of the vaccine, if considered relevant by the investigator.
5. Contraindication for MRI imaging such as metallic endoprosthesis or stent implantation in the last 6 months.
6. Any of the following detected by brain MRI:

* Thromboembolic infarction

* Other focal lesions which may be responsible for the cognitive status of the patient such as infectious disease, space-occupying lesions, normal pressure hydrocephalus or any other abnormalities associated with significant central nervous disease other than Alzheimer’s disease.

* More than one lacunar infarct defined as a focal lesion of CSF signal intensity with a diameter of less than 1.5 cm in any dimension

* Any lacunar infarct in a strategically important location such as the thalamus, hippocampus of either hemisphere, head of the left caudate

* White matter lesions involving more than 25 % of the hemispheric white matter

1. Surgery (under general anaesthesia) within 3 months prior to study entry and scheduled surgery during the whole study period.
2. History and/or presence of autoimmune disease, if considered relevant by the investigator.
3. Recent (≤ 3 years since last specific treatment) history of cancer (Exceptions: basal cell carcinoma, intraepithelial cervical neoplasia).
4. Active infectious disease (e.g., Hepatitis B, C).
5. Presence and/or history of Immunodeficiency (e.g., HIV).
6. Significant systemic illness (e.g., chronic renal failure, chronic liver disease, poorly controlled diabetes, poorly controlled congestive heart failure, congenital long QT syndrome, other deficiencies), if considered relevant by the investigator.
7. Hypothyroidism, defined as any significant thyroid-stimulating hormone elevation. Patients with corrected hypothyroidism are eligible for the study provided that treatment has been stable for 3 months before study entry.
8. History of significant psychiatric illness such as schizophrenia, bipolar affective disorder or major depression.
9. Current depressive episode (Geriatric Depression Scale GDS > 5 at Visit 1).
10. Metabolic or toxic encephalopathy or dementia due to a general medical condition.
11. Alcoholism or substance abuse within the past year (alcohol or drug intoxication).
12. Wernicke’s encephalopathy
13. History or evidence of any other CNS disorder that could be the cause of dementia (infectious or inflammatory/demyelinating CNS conditions, Creutzfeldt-Jakob disease, Parkinson’s disease, Huntington’s disease, brain tumour, subdural haematoma, etc.)
14. History or evidence of cerebrovascular disease (ischemic or haemorrhagic stroke, transient ischemic attack), or diagnosis of possible, probable or definite vascular dementia.
15. Epilepsy.
16. Prior and/or current treatment with experimental immunotherapeutics including IVIG or any vaccines for AD.
17. Current treatment with immunosuppressive drugs.
18. Change in dose of standard treatments for AD or hypothyroidism within 3 months prior to Visit 1.
19. Change in dose of previous and current medications which the patient is taking because of consisting illnesses according medical history within the last 30 days prior to Visit 1, if considered clinically relevant by the investigator.

# Inclusion and exclusion criteria of the AC-AD-002 follow up study

The inclusion and exclusion criteria of the present study are replicated verbatim below. Please note that the study approached only patients of the preceding trial for enrolment, thus other aspects of patient suitability (diagnosis, etc.) were already established previously.

## Inclusion criteria (AC-AD-002)

The subjects have to meet the following criteria upon enrolment:

1. Completion of visit V8 of the AADvac1 phase I study Axon CO 18700 (EUDRACT 2012-003916-29).
2. Informed consent capability (as determined by an independent neurologist/psychiatrist).
3. Written informed consent signed and dated by the patient and the caregiver.
4. Availability of a partner/caregiver knowing the patient and being able to accompany the patient to the visits
5. Adequate visual and auditory abilities and language skills to allow neuropsychological testing.
6. Female patients are only eligible for the study if they are either surgically sterile or at least 2 years postmenopausal.
7. Sexually active males must be using reliable contraception methods (i.e. condoms) or be surgically sterile.

## Exclusion criteria (AC-AD-002)

Subjects presenting with any of the following upon enrolment will not be included into the study:

1. Pregnant or breastfeeding women.
2. Participation in another clinical trial during the course of this study.
3. Contraindication for MRI imaging such as MRI-incompatible metallic endoprosthesis or MRI-incompatible stent implantation
4. History and/or presence of autoimmune disease, if considered relevant by the investigator.
5. Significant systemic illness (e.g., chronic renal failure, chronic liver disease, poorly controlled diabetes, poorly controlled congestive heart failure, congenital long QT syndrome, other deficiencies), if considered relevant by the investigator.
6. Current treatment with immunosuppressive drugs.

# Subject disposition


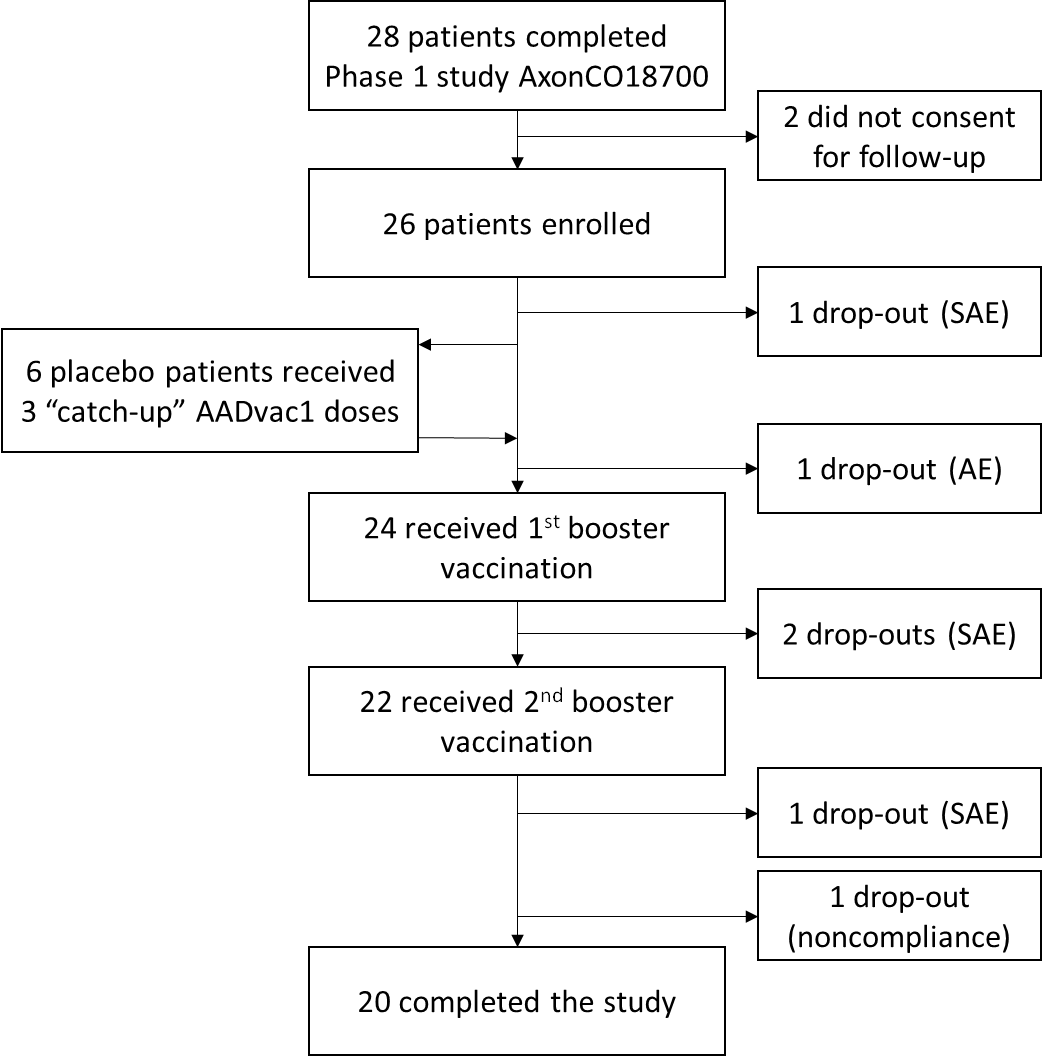


**Figure 3: Subject disposition**

# Histological comparison of AADvac1-induced antibodies to reference antibodies

Staining of AD brain tissue with the serum of an AADvac1-treated patient was compared to positive controls - AADvac2 antibody (AX004, conformational), AT8 (pS202/pT205, phospho-specific), and negative control (pre-treatment serum).


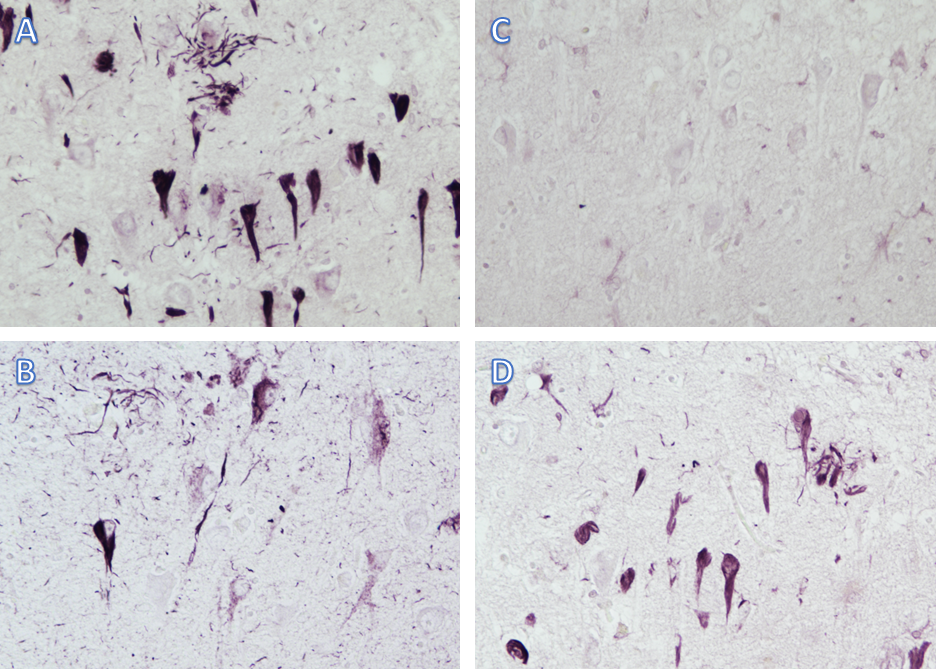


**Figure 4: AADvac1 induces antibodies that label all aspects of neurofibrillary pathology.** A) AADvac2 antibody (AX004, positive control). B) AT8 anti-phospho-tau pS202/pT205 (positive control). C) pre-treatment serum (negative control). D) serum of patient treated with 6 doses of AADvac1.

# Histology (non-demented control brain)


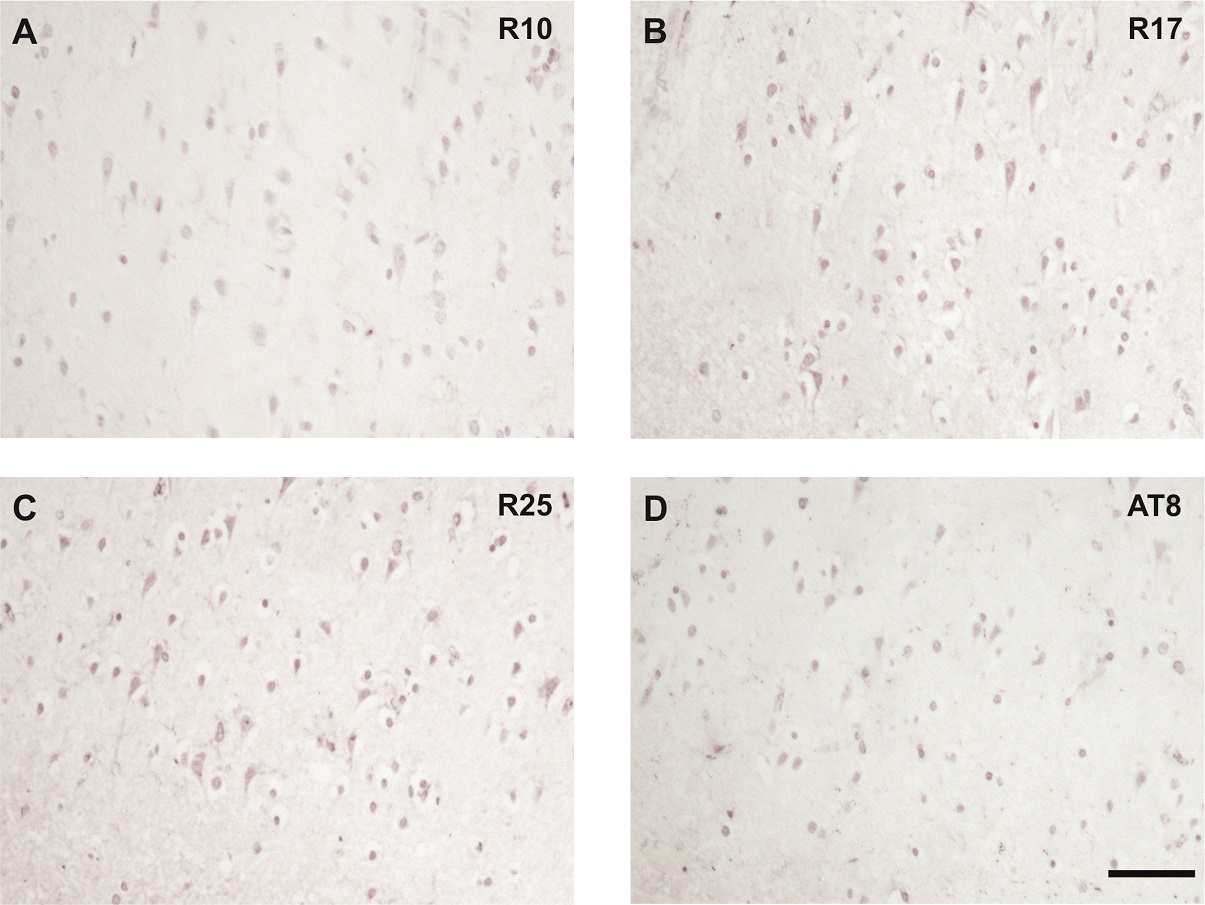


**Figure 5: Sera of AADvac1-treated patients don't label non-demented control tissue.** Scale bar 100 μm. Patient R17 had an anti-AD tau titre of 1:30999: R25 a titre of 1:18185; and R10 a titre of 1:12800. AT8 staining shown as control.

# Staining of AD brain extracts with AADvac1-induced antibodies


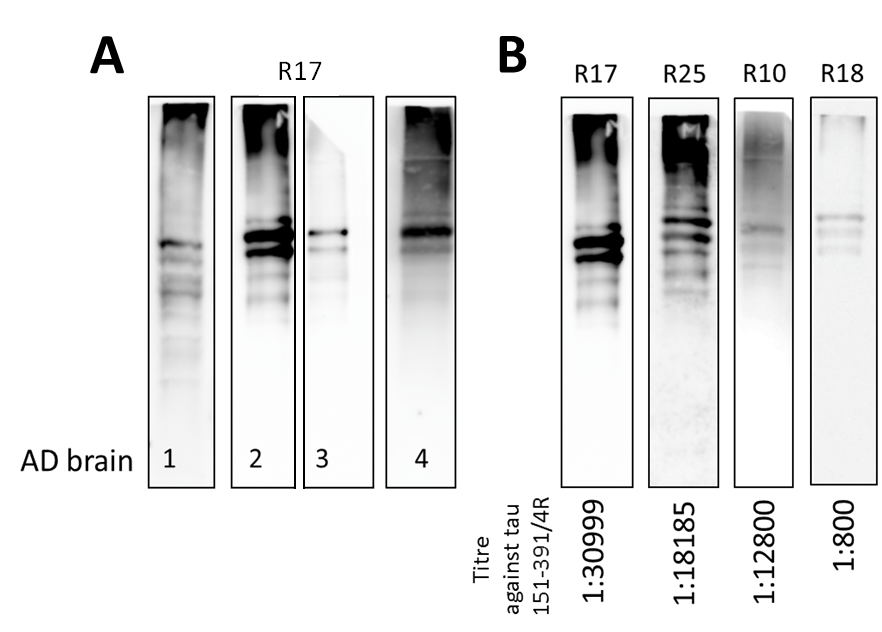


**Figure 6: Sera of selected patients treated with AADvac1 detect high- and low-molecular-weight pathological tau species** in all assessed brain extracts (A). Sera from different AADvac1-treated patients detect the same AD brain extract. Intensity of the staining is proportional to the antibody titre generated by the patients against pathological tau (B).

# CSF anti-peptide response vs. serum anti-peptide response

Figure 7: Titres of AADvac1-induced antibodies in the CSF correlate with those in peripheral blood

# MRI settings

MRI scans were performed at screening, week 4, week 12, week 16 and week 24. The screening scan served as baseline.

The MRI scans performed at each assessment included:

- one or two combined scout scans (T1 weighted axial scout, T1 weighted coronal scout, T1 weighted sagittal scout)
- an axial T2 weighted sequence
- an axial FLAIR sequence
- an axial T2* weighted scan
- a sagittal T1 weighted 3D scan
- an optional diffusion-weighted sequence

The scout scans served for exact repositioning in the follow-up examinations. The FLAIR and T2 weighted scans served to assess white matter changes and to check for conformity to inclusion criteria. The T2* weighted gradient sequence served to detect microbleeds. The sagittal 3D T1 weighted scan was used for assessing brain atrophy including loss of hippocampal, cortical and whole brain volume.

Table 1: Sequence parameters for 3 Tesla scanners

|  | **axial T2w** | **axial FLAIR** | **Axial T_2_*** | **sagittal 3DT1*** |
| --- | --- | --- | --- | --- |
| **Method** | Fast spin echo | IR-fast spin echo | Spoiled gradient echo | IR-prepped fast 3D gradient echo (spoiled) |
| **FOV (mm)** | 240 | 240 | 240 | 256 |
| **Rectangular FOV** | 6/8 or 70% | 6/8 or 70% | 6/8 or 70% | 100% |
| **foldover direction**  **(phase encoding)** | R→L | R→L | R→L | A→P |
| **Acq. Matrix** | 256x256 | 256x256 | 256x256 | 256x256 |
| **Slice thickness (mm)** | 5 | 5 | 5 | 1.0 |
| **Slice gap (mm)** | 0 | 0 | 0 | 0 |
| **# Slices**** | 24 -28 | 24 - 28 | 24 - 28 | 170 |
| **TR (ms)** | 4000 - 6000 | 9000 - 10000 | 600 | 1800^†^  shortest^††^  5-6^†††^ |
| **TE (ms)** | 90-100 | 90-100 | 20 | minimum |
| **TI (ms)** | X | 2200 – 2400,  2800^††^ | X | 900^†,††^  450^†††^ |
| **Flip angle (°)** | 90 | 90 | 20 | 8 - 12 |
| **Echo train length**  **(turbo factor)** | 8 - 10 | 8 - 10 | X | X |
| **Averages (NEX)** | variable | variable | 1 | 1 |
| **Saturation slab** | no | inferior | no | no |
|  |  |  |  |  |
| ***~ Duration (min.)*** | 3 - 4 | 4 - 6 | 4 | 6-7 |

*† for Siemens, †† for Philips, ††† for GE*

**Alternatively, a pre-set T1 weighted 3D scan provided by the manufacturer could be used as long as the same effective resolution and contrast to noise ratio was achieved. The appropriateness of the sequence was checked during the certification scan phase.*

*For Philips: 3D IR-TFE sequence (check pre-set procedures for optimized sequence); for Siemens: MPRAGE sequence; for GE: Fast IR prepped 3D SPGR.*

***Number of slices was adjusted to provide whole brain coverage*

*Note: a more detailed protocol was provided for each centre based on the results of a technical survey.*

Table 2: Sequence parameters for 1.5 Tesla scanners

|  | **axial T2w** | **axial FLAIR** | **Axial T_2_*** | **sagittal 3DT1*** |
| --- | --- | --- | --- | --- |
| **Method** | Fast spin echo | IR-fast spin echo | Spoiled gradient echo | IR-prepped fast 3D gradient echo (spoiled) |
| **FOV (mm)** | 240 | 240 | 240 | 256 |
| **Rectangular FOV** | 6/8 or 70% | 6/8 or 70% | 6/8 or 70% | 100% |
| **foldover direction**  **(phase encoding)** | R→L | R→L | R→L | A→P |
| **Acq. Matrix** | 256x256 | 256x256 | 256x256 | 256x256 |
| **Slice thickness (mm)** | 5 | 5 | 5 | 1.0 |
| **Slice gap (mm)** | 0 | 0 | 0 | 0 |
| **# Slices**** | 24 -28 | 24 - 28 | 24 - 28 | 170 |
| **TR (ms)** | 4000 - 6000 | 9000 - 10000 | 600 | 2400^†^  shortest^††^  9-14^†††^ |
| **TE (ms)** | 120 | 120 | 30 | minimum |
| **TI (ms)** | X | 2200  2500^††^ | X | 1000^†,††^  450^†††^ |
| **Flip angle (°)** | 90 | 90 | 25 | 8 - 12 |
| **Echo train length**  **(turbo factor)** | 8 - 10 | 8 - 10 | X | X |
| **Averages (NEX)** | variable | variable | 1 | 1 |
| **Saturation slab** | no | inferior | no | no |
|  |  |  |  |  |
| ***Duration (min.)*** | 3 - 4 | 4 - 6 | 4 | 7-8 |

*† for Siemens, †† for Philips, ††† for GE*

**Alternatively, a pre-set T1 weighted 3D scan provided by the manufacturer could be used as long as the same effective resolution and contrast to noise ratio was achieved. The appropriateness of the sequence was checked during the certification scan phase.*

*For Philips: 3D IR-TFE sequence (check pre-set procedures for optimized sequence); for Siemens: MPRAGE sequence; for GE: Fast IR prepped 3D SPGR.*

***Number of slices was adjusted to provide whole brain coverage*

Table 3: Axial diffusion weighted sequence

| **axial diffusion weighted sequence for 1.5T and 3T** | |
| --- | --- |
| Method | Single shot  spin echo - echo planar imaging  EPI |
| FOV (mm) | 240 |
| Rectangular FOV | 6/8 or 70% |
| foldover direction  (phase encoding) | A->P |
| Acq. Matrix | 128x128 |
| Slice thickness (mm) | 3 |
| Slice gap (mm) | 0 |
| # Slices** | 42-46 |
| TR (ms) | 6000 |
| TE (ms) | shortest |
| Flip angle (°) | 90 |
| b-values (s/mm^2)^ | 0/1000 |
| Number of directions | 12 |
| Averages (NEX) | 4 |
| Saturation slab | no |
| Fast suppression | yes |
| *Duration (min.)* | 3 - 4 |

# Longitudinal change on ADAS-Cog, CFT, LFT, and MMSE


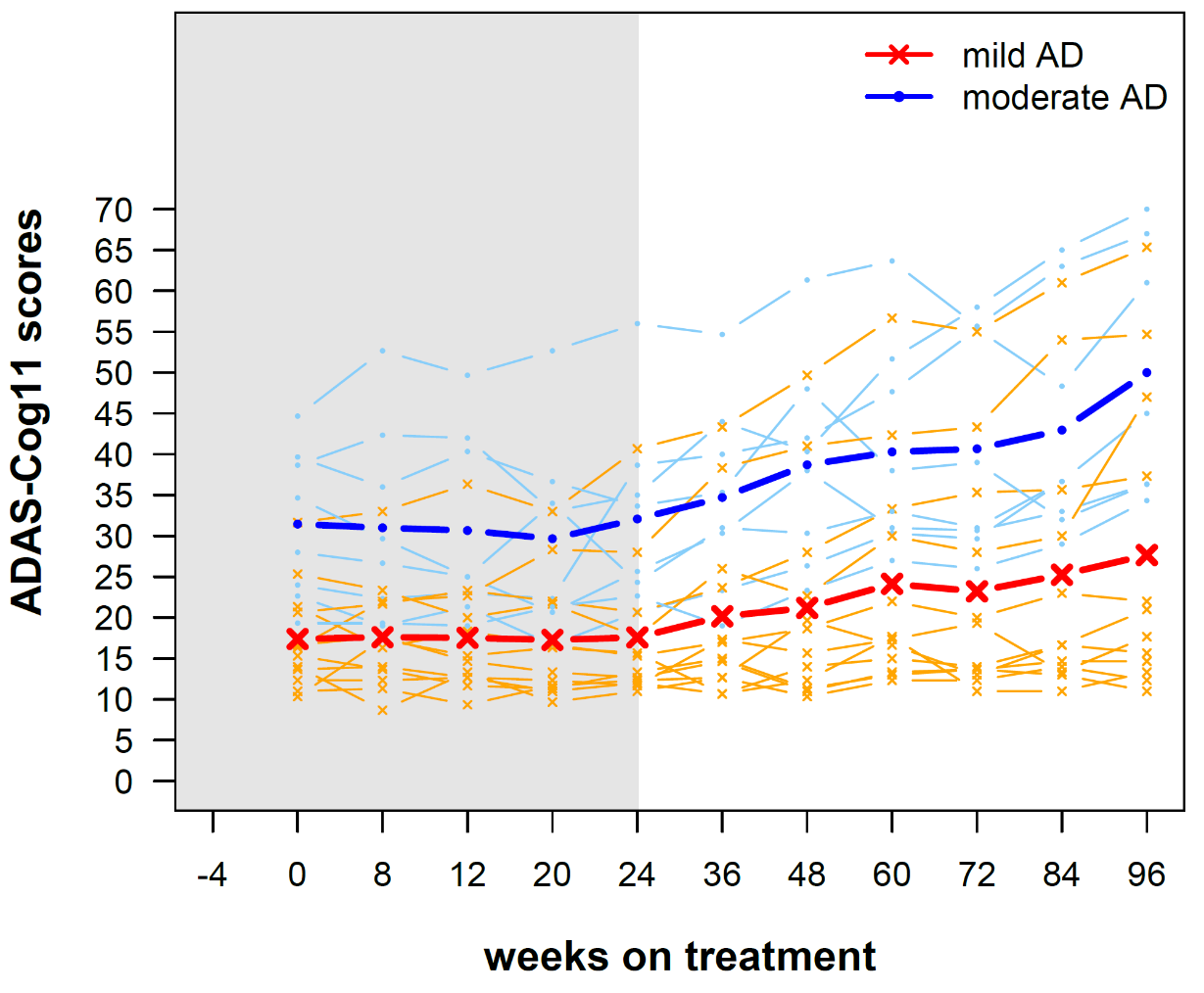


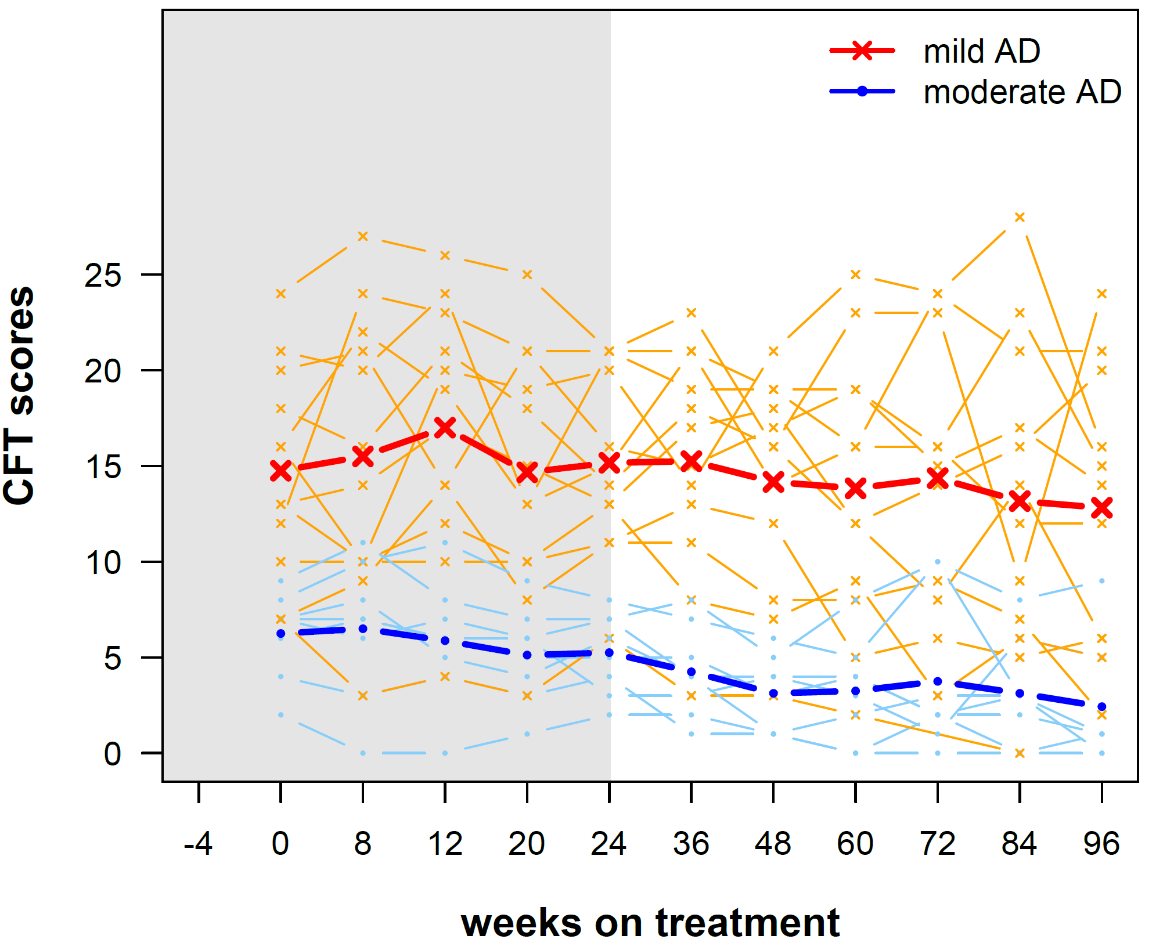


**
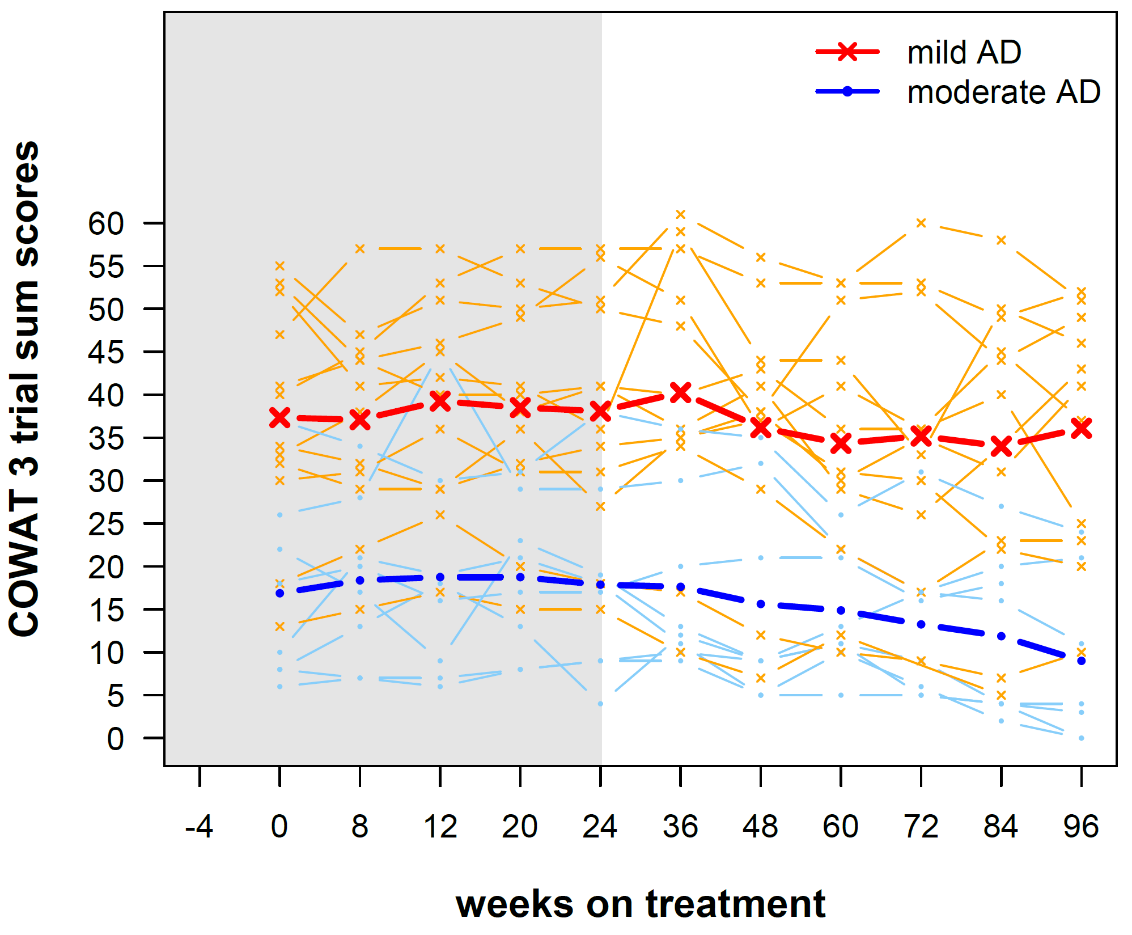
**

**
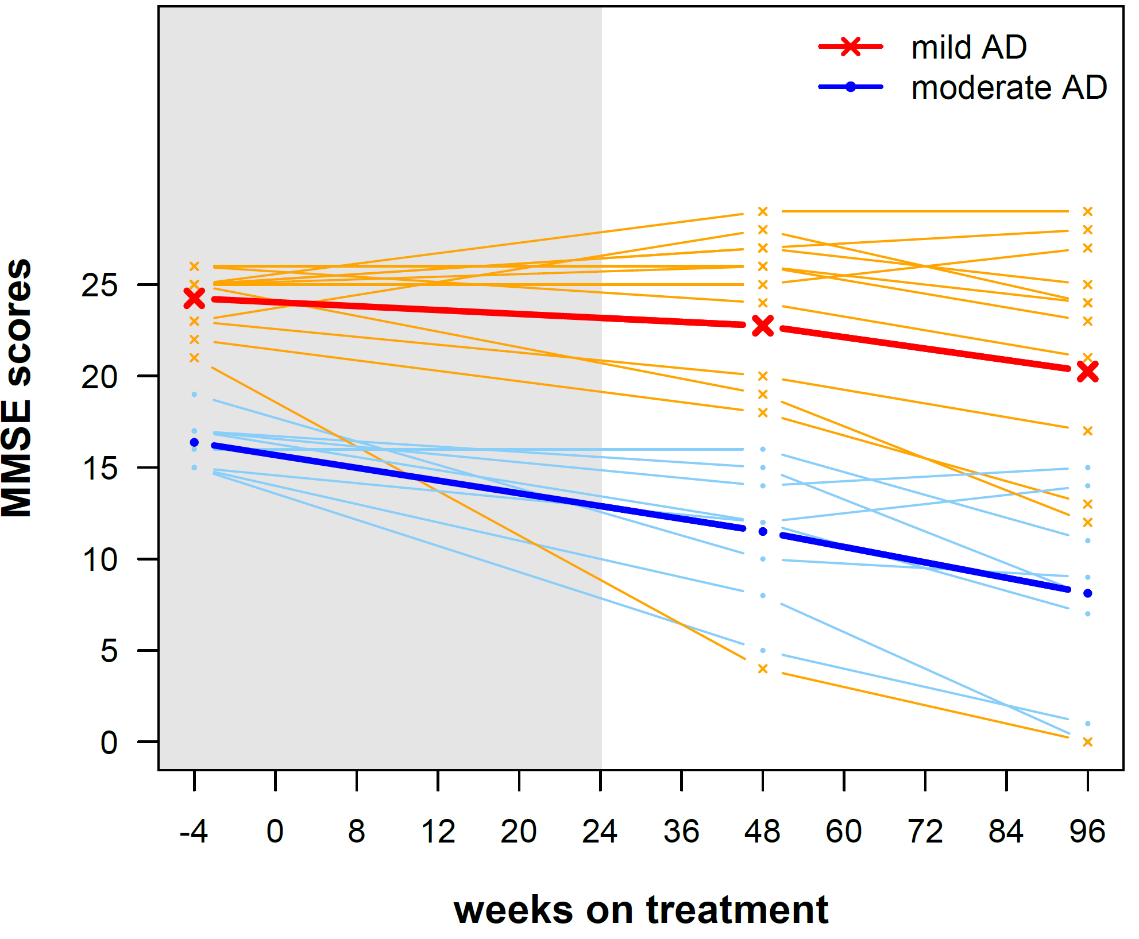
**

**Figure 8: Patient cognition over 96 weeks on AADvac1 treatment, displayed as absolute change.** Values shown separately for patients with mild AD (MMSE 20-26, red/orange) and moderate AD (MMSE 15-19, blue) at treatment start. Thick lines indicate mean cognitive change, thin lines the courses of individual patients. For ADAS-Cog11, higher scores indicate greater impairment. For the other tests, higher scores mean better cognitive function. Values obtained in preceding phase 1 study "Axon CO18700" indicated by grey background.

# IgG titre AUC vs. brain atrophy

A) (n.s.)

B) (n.s.)

C) (n.s.)

D) (n.s.)

E) (r = 0·544, p = 0·0196)

F) (r = 0·476, p = 0·0460)

G) (n.s.)

H) (n.s.)

I) (n.s.)

J) (n.s.)

K) (r = 0·683, p = 0·050)

L) (r = 0·750, p = 0·025)

Figure 9: Correlation plots of IgG AUC of titres against Axon Peptide 108 and hippocampal atrophy. Atrophy rates shown in % of baseline volume change.

Spearman's r correlation coefficient shown. Hippocampal volume correlations replicated from main article body for the sake of completeness.

# Longitudinal brain atrophy


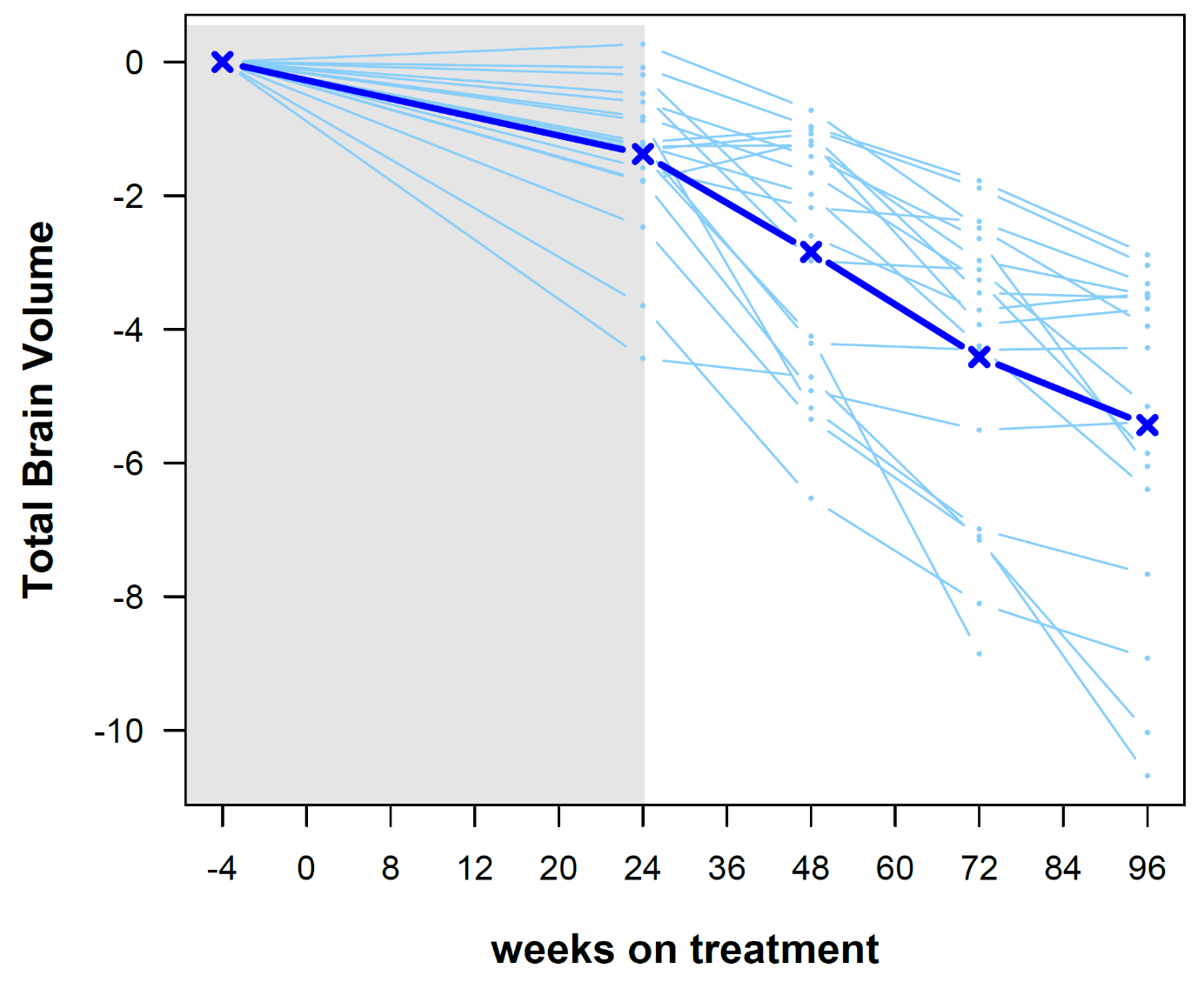


Figure 10: Change in total brain volume over 96 weeks of treatment (in %)

| TBV | 24w | 48w | 72w | 96w |
| --- | --- | --- | --- | --- |
| Mean | -1.377 | -2.843 | -4.415 | -5.433 |
| Std. Deviation | 1.152 | 1.842 | 2.198 | 2.448 |

*Values shown as % change.*

*
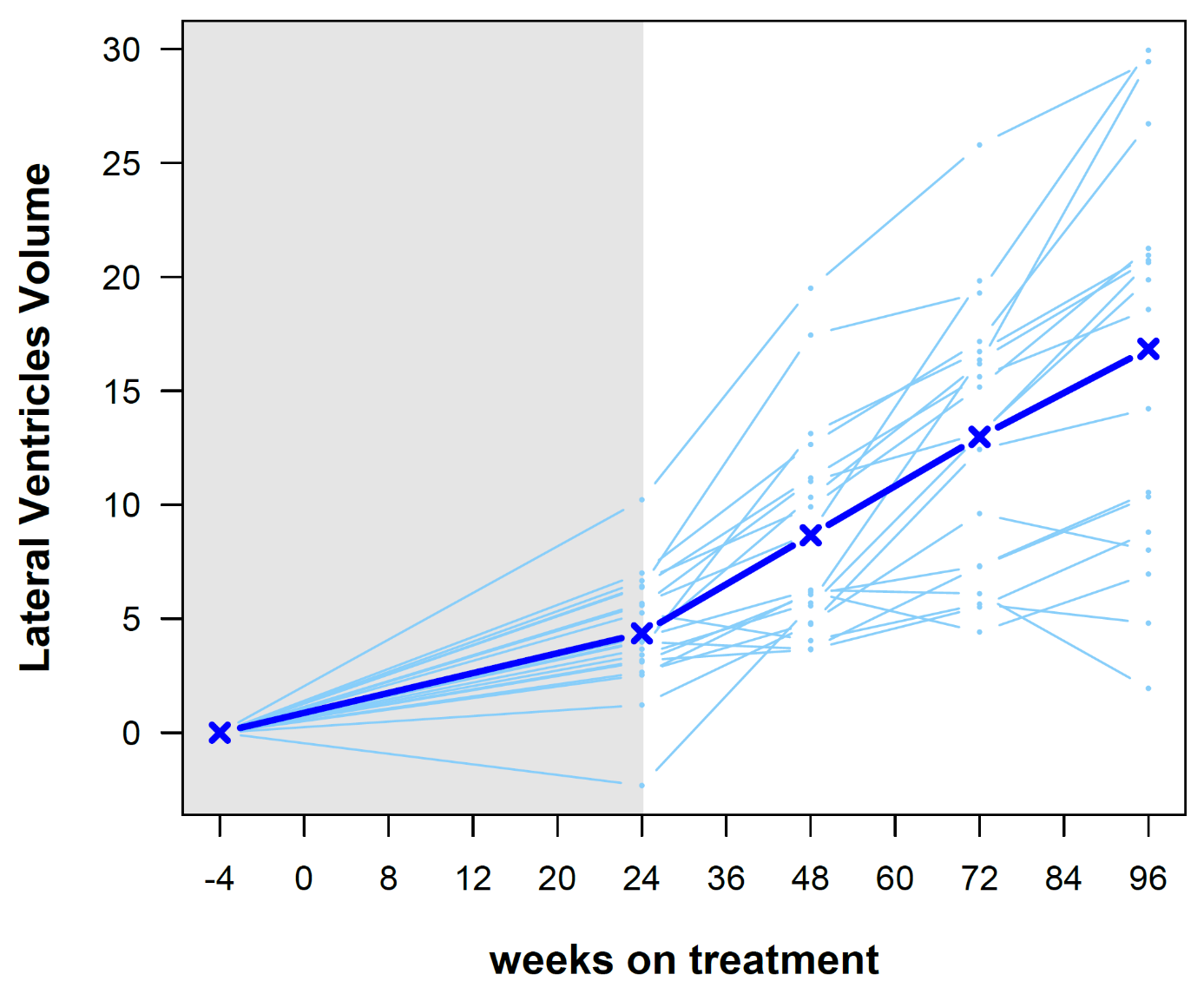
*

Figure 11: Change in lateral ventricle volume over 96 weeks of treatment (in %)

| LVV | 24w | 48w | 72w | 96w |
| --- | --- | --- | --- | --- |
| Mean | 4.36 | 8.664 | 12.98 | 16.84 |
| Std. Deviation | 2.629 | 4.627 | 5.88 | 8.946 |

*Values shown as % change.*

*
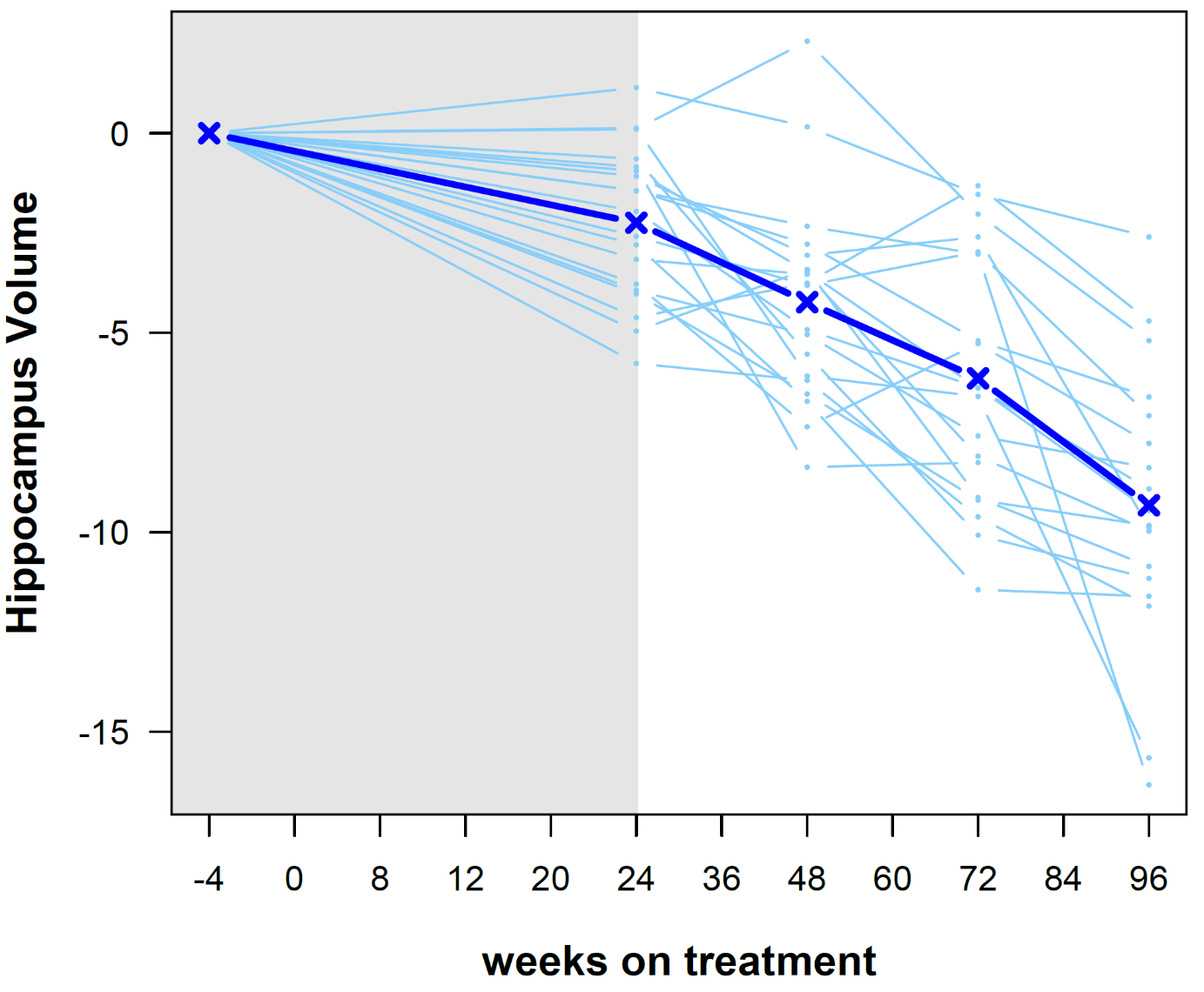
*

Figure 12: Change in hippocampus volume over 96 weeks of treatment (in %, sum of left and right hippocampus)

| HCV | 24w | 48w | 72w | 96w |
| --- | --- | --- | --- | --- |
| Mean | -2.243 | -4.234 | -6.141 | -9.324 |
| Std. Deviation | 1.923 | 2.579 | 3.161 | 3.488 |

*Values shown as % change.*

# AADvac1-induced antibody titres vs. NFT counts (mice)

r = -0·860, p = 0·006 r = -0·747, p = 0·033 r = -0·921, p = 0·003

Figure 13: A pronounced negative correlation between AADvac1-induced antibody titres and NFT numbers in transgenic animals
